# HTLV-1 Hbz protein, but not *hbz* mRNA secondary structure, is critical for viral persistence and disease development

**DOI:** 10.1371/journal.ppat.1011459

**Published:** 2023-06-16

**Authors:** Victoria Maksimova, Tasha Wilkie, Susan Smith, Cameron Phelps, Corrine Melvin, Lianbo Yu, Stefan Niewiesk, Patrick L. Green, Amanda R. Panfil

**Affiliations:** 1 Center for Retrovirus Research, Department of Veterinary Biosciences, College of Veterinary Medicine, The Ohio State University, Columbus, Ohio, United States; 2 Department of Biomedical Informatics, College of Medicine, The Ohio State University, Columbus, Ohio, United States; 3 Comprehensive Cancer Center and Solove Research Institute, The Ohio State University, Columbus, Ohio, United States; Imperial College London Faculty of Medicine, UNITED KINGDOM

## Abstract

Human T-cell leukemia virus type 1 (HTLV-1) is the etiologic cause of adult T-cell leukemia/lymphoma (ATL) and encodes a viral oncoprotein (Hbz) that is consistently expressed in asymptomatic carriers and ATL patients, suggesting its importance in the development and maintenance of HTLV-1 leukemic cells. Our previous work found Hbz protein is dispensable for virus-mediated T-cell immortalization but enhances viral persistence. We and others have also shown that *hbz* mRNA promotes T-cell proliferation. In our current studies, we evaluated the role of *hbz* mRNA on HTLV-1-mediated immortalization *in vitro* as well as *in vivo* persistence and disease development. We generated mutant proviral clones to examine the individual contributions of *hbz* mRNA, *hbz* mRNA secondary structure (stem-loop), and Hbz protein. Wild-type (WT) and all mutant viruses produced virions and immortalized T-cells *in vitro*. Viral persistence and disease development were also evaluated *in vivo* by infection of a rabbit model and humanized immune system (HIS) mice, respectively. Proviral load and sense and antisense viral gene expression were significantly lower in rabbits infected with mutant viruses lacking Hbz protein compared to WT or virus with an altered *hbz* mRNA stem-loop (M3 mutant). HIS mice infected with Hbz protein-deficient viruses showed significantly increased survival times compared to animals infected with WT or M3 mutant virus. Altered *hbz* mRNA secondary structure, or loss of *hbz* mRNA or protein, has no significant effect on T-cell immortalization induced by HTLV-1 *in vitro*; however, the Hbz protein plays a critical role in establishing viral persistence and leukemogenesis *in vivo*.

## Author summary

Human T-cell leukemia virus type 1 (HTLV-1) is the cause of an aggressive malignancy called adult T-cell leukemia/lymphoma. HTLV-1 encodes a viral oncoprotein (Hbz) that is consistently expressed in patients, suggesting its importance in the development and maintenance of leukemic cells. Remarkably, the *Hbz* gene is bimodal, with functions at both the mRNA and protein levels. In our study, we generated mutant proviral clones to examine the individual contributions of *hbz* mRNA, *hbz* mRNA secondary structure (stem-loop), and Hbz protein. Viral persistence and disease development were evaluated *in vivo* by infection of a rabbit model and humanized immune system (HIS) mice, respectively. Proviral load and viral gene expression were significantly lower in rabbits infected with mutant viruses lacking Hbz protein compared to WT or virus with an altered *hbz* mRNA stem-loop (M3 mutant). HIS mice infected with Hbz protein-deficient viruses showed significantly increased survival times compared to animals infected with WT or M3 mutant virus. Our data demonstrate that, despite the structure-dependent function that *hbz* mRNA exerts to stimulate proliferation of cell lines, alteration of a single 5’ stem-loop structure of the RNA alone has no effect on the ability of HTLV-1 to persist and induce leukemogenesis *in vivo*.

## Introduction

Human T-cell leukemia virus type 1 (HTLV-1) infection is linked to the development of adult T-cell leukemia/lymphoma (ATL) [1–3], an aggressive CD4^+^ T-cell malignancy. Although HTLV-1 establishes a lifelong persistence and primarily replicates by driving the proliferation of infected cells, there is a chronic immune response against viral proteins, and disease develops in a small percentage of infected carriers. Risk factors, including age, proviral load, and genetic or epigenetic markers, as well as the mechanisms underlying HTLV-1-induced disease continue to be elucidated.

The sense strand of the HTLV-1 genome encodes the *gag*, *pro*, *pol*, and *env* structural and enzymatic genes common to retroviruses, as well as regulatory (*tax*, *rex*) and accessory genes (*p12/p8*, *p13*, *p30*) [4]. In addition, HTLV-1 produces mRNA and protein products from a gene, *hbz*, on the antisense strand of the proviral genome [5, 6]. *Hbz* is expressed from a TATA-less promoter in the 3’ LTR and is regulated by Sp1 binding sites located in the promoter region [7–11]. Hbz competes for interaction with the same cellular factors as Tax (e.g., CBP/p300), thus, down-regulating Tax-mediated sense transcription from the 5’ LTR [12]. Hbz also disrupts members of the AP-1 family by inhibiting c-Jun and JunB DNA-binding activities [13, 14] and interacting with JunD to promote expression of its own mRNA [10]. In addition, it has been shown that Hbz protein interacts with the retinoblastoma (Rb)/E2F-1 complex to promote cell cycle progression and apoptosis.[15] *Hbz* is frequently the only viral transcript expressed in ATL cells [7, 12, 16], and the 3’ region of the provirus remains transcriptionally active due to the presence of a viral CTCF binding site [17] and an internal enhancer element [18]. These factors point to key roles for Hbz in the maintenance of infected cells and leukemogenic progression.

The role of the Hbz protein has been characterized within the context of full-length HTLV-1 to assess its contribution to early infection events. Hbz was previously shown to be dispensable for HTLV-1-induced T-cell immortalization *in vitro*; however, loss of the protein leads to reduced viral infectivity in rabbits [19]. Experiments using short hairpin RNAs have also demonstrated that the knockdown of both *hbz* mRNA and protein decreases the proliferation of the HTLV-1-transformed T-cell line, SLB-1 [20]. Subsequent inoculation of these Hbz-deficient cells into NOD/SCID^γchain-/-^ mice led to decreased proliferation, tumor formation, and organ infiltration compared to mice that received wild-type (WT) cells [20]. In addition, it was found that spliced *hbz* is the primary transcript expressed in ATL cells and shRNA-mediated inhibition of *hbz* reduced cell proliferation [7]. Further experiments to dissect the roles of *hbz* mRNA and protein were performed in Kit-225 cells (HTLV-1-negative T-cell line) using Hbz expression vectors, which showed that the *hbz* mRNA conferred growth-promoting effects [7]. Silent mutations that completely disrupted the secondary structure of the *hbz* mRNA also abrogated the proliferative effect and silent RNA mutations suggested a structure-dependent mechanism [7].

Here we addressed the hypothesis that Hbz protein and mRNA have distinct roles in viral persistence and pathogenesis using full-length infectious molecular clones *in vitro* and *in vivo*.

## Materials and methods

### Ethics statement

All animal procedures were performed in accordance with a protocol approved by University Laboratory Animal Resources (ULAR) of The Ohio State University. The animal use protocol received prior approval by the Institutional Animal Care and Use Committee (IACUC) of The Ohio State University.

### Cell culture

Human embryonic kidney (HEK) 293T cells were cultured in Dulbecco’s modified Eagle’s medium (DMEM) (Gibco, Thermo Fisher Scientific, Waltham, MA). All cells and cell lines were cultured in media containing 10% fetal bovine serum (FBS), 100 U/mL penicillin, 100 μg/mL streptomycin, and 2 mM L-glutamine and maintained in a humidified atmosphere of 5% CO_2_ and air at 37°C, except where noted. Parental human 729.B control cells (lymphoblastoid B-cell line) and 729 HTLV-1 producer cell lines were cultured in Iscove’s DMEM (Mediatech, Inc. Manassas, VA). Human PBMCs (hPBMCs) were isolated from whole blood freshly collected from healthy donors using Ficoll-Paque PLUS (Cytiva, Marlborough, MA). Protocols for blood collection from human donors were approved by the Ohio State University Institutional Review Board. Formal written consent from human donors was obtained prior to blood collection. hPBMCs and early passage, HTLV-1-immortalized primary human T-cell lines (PBLs) were cultured in Roswell Park Memorial Institute (RPMI) 1640 medium (Gibco, Thermo Fisher Scientific, Waltham, MA) supplemented with 20% FBS and 10 U/mL recombinant human interleukin-2 (hIL-2; Roche Diagnostics GmbH, Mannheim, Germany).

### Plasmids and cloning

The infectious HTLV-1 proviral clone ACHneo (WT) has been previously characterized [21, 22]. *Hbz* mRNA and protein mutants in Table 1 were generated by subcloning, site-directed mutagenesis, and ligation of the mutated regions back into the full-length HTLV-1 proviral plasmid. The Hbz M3 mutant contains a single C to T point mutation at nucleotide 11 of the spliced *hbz* transcript that disrupts a stem-loop secondary structure of the RNA [7]. The ΔHbz mutant contains a G to A point mutation which terminates the Hbz reading frame at amino acid 8 of the spliced, major transcript, but allows for *hbz* mRNA production [19]. The M3.ΔHbz mutant contains the combined M3 and ΔHbz mutations, resulting in the production of *hbz* mRNA with altered secondary structure and truncated Hbz protein. Lastly, the Hbz splice acceptor mutant (SAm) contains a T to G point mutation of the *hbz* splice acceptor site [23]. The SAm mutation prevents *hbz* mRNA splicing and protein production. The HTLV-1 5’LTR-luciferase reporter plasmid (LTR-1-Luc) was described previously [24]. The pcDNA3.1^(+)^ negative control (empty vector) was purchased from Invitrogen (Carlsbad, CA).

**Table 1:** Descriptions of Hbz variant proviral clones. *Hbz* mRNA and protein mutants were generated using the HTLV-1 wild-type (WT) molecular clone ACH. Mutations were designed to disrupt RNA splicing, RNA secondary structure, or protein production of Hbz, as described.

Proviral Clone	Mutation	*hbz* mRNA	HBZ protein
WT	None	+	+
M3	Point mutation: 11^th^ nucleotide	Altered stem-loop	+
ΔHbz	Stop codon: 8^th^ amino acid	+	-
M3.ΔHbz	Both M3 and ΔHbz mutations	Altered stem-loop	-
SAm	Point mutation: splice acceptor site	-	-

### Transfections, luciferase reporter assays, and p19 Gag ELISA

HEK293T were co-transfected with empty vector or proviral plasmid DNA and LTR-1-Luc using *Trans*IT-2020 Transfection Reagent (Mirus Bio LLC, Madison, WI) according to the manufacturer’s protocol. 48h post-transfection, cell supernatants were collected to measure HTLV-1 p19 Gag production using the RETRO-TEK HTLV p19 Antigen ELISA (ZeptoMetrix Corporation, Buffalo, NY). Luciferase assays were performed by lysing cell pellets in Passive Lysis Buffer (Promega, Madison, WI) and following the manufacturer’s protocol for the Luciferase Assay System (Promega, Madison, WI). Firefly luciferase relative light units were measured using the FilterMax F5 Multi-Mode Microplate Reader (Molecular Devices, San Jose, CA).

### Generation of HTLV-1 producer cells

Stable producer cell lines were generated by nucleofecting 729.B cells with 2 μg WT, M3, ΔHbz, M3.ΔHbz, or SAm proviral plasmid DNA (each containing a neomycin resistance gene) using the Amaxa Cell Line Nucleofector Kit V and Nucleofector2b Device (program X-001) according to the manufacturer’s protocol (Lonza Cologne AG, Cologne, Germany). 72h post-transfection, cells were placed under 1 mg/mL G418 selection (Gibco, Thermo Fisher Scientific, Waltham, MA). Approximately two weeks after G418 selection, cell supernatant was collected to measure HTLV-1 p19 Gag production by ELISA (ZeptoMetrix Corporation, Buffalo, NY). Polyclonal cells with confirmed p19 production were subjected to single cell dilution. Single cell clones were plated at a density of 1 x 10^6^ cells in 2mL media and supernatant was collected after 24h for ELISA to obtain normalized p19 Gag values. 729 mutant producer cells with p19 Gag production comparable to established 729.WT producer cells were used in subsequent experiments. Point mutations within the *hbz* gene for each mutant were verified in genomic DNA by PCR and Sanger sequencing.

### Immunoblotting

Transfected HEK293T cells and 729.B parental, WT, or mutant producer cell clones were lysed in NP-40 buffer (150 mM NaCl, 50 mM Tris-HCl [pH 8.0], and 1% NP-40) with protease inhibitor cocktail (cOmplete, Mini Protease Inhibitor Cocktail, Roche Diagnostics GmbH, Mannheim, Germany). Total protein was quantitated using the Pierce BCA Protein Assay Kit (Thermo Fisher Scientific, Rockford, IL) and FilterMax F5 Multi-Mode Microplate Reader (Molecular Devices, San Jose, CA). Protein (10 μg) was loaded in equal amounts on 4–20% Mini-PROTEAN TGX Precast Protein Gels (Bio- Rad, Hercules, CA) and transferred onto Amersham Protran Western blotting nitrocellulose membranes (Cytiva, Marlborough, MA). Membranes were blocked with 5% milk in PBS with 0.1% Tween-20 and incubated with anti-Hbz (rabbit anti-sera [19]; 1: 500) and anti-β-actin (1:5,000; A2228, Sigma-Aldrich, St. Louis, MO). Membranes were incubated in appropriate anti-rabbit or -mouse IgG (H+L) HRP conjugate secondary antibodies (1:5,000; W401B or W402B, Promega, Madison, WI). Pierce ECL Western Blotting Substrate (Thermo Fisher Scientific, Rockford, IL) was used to develop the membranes and images were captured using an Amersham Imager 600 (GE Healthcare Life Sciences).

### *In vitro* immortalization assay

Approximately 2 x 10^6^ freshly isolated hPBMCs from two healthy donors were co-cultured independently with 1x 10^6^ lethally irradiated (100 Gy) 729.B parental cells, 729.WT, 729.M3, 729.ΔHbz, 729.M3.ΔHbz, or 729.SAm producer cells in 24-well dishes. 10 U/mL hIL-2 was provided weekly when media was refreshed. A portion of each irradiated producer cell line was maintained in culture to verify cell death. Viable cell counts were determined at weekly intervals in triplicate wells for each condition by Trypan Blue exclusion (Gibco, Thermo Fisher Scientific, Waltham, MA). Culture supernatant was collected from the enumerated wells to measure p19 Gag production using the RETRO-TEK HTLV p19 Antigen ELISA (ZeptoMetrix Corporation, Buffalo, NY). Immortalization results shown are representative of two independent experiments. Newly immortalized PBL cell lines from a representative blood donor were utilized for subsequent in vitro characterization experiments. The number of cell lines generated in the WT and Hbz mutant conditions are as follows: WT n = 4, M3 n = 2, ΔHbz n = 3, M3.ΔHbz n = 3, and SAm n = 3.

Immortalized PBL cell lines were collected by slow centrifugation (5 min, 800 x *g*) for phenotypic analysis. Collected cells were stained using fluorescein isothiocyanate (FITC)-conjugated anti-human CD3 and phycoerythrin (PE)-conjugated anti-human CD4 or CD8 antibodies (BD Biosciences, San Jose, CA) to evaluate percentages of CD4^+^ and CD8^+^ T-cells using a Guava easyCyte Benchtop Flow Cytometer. The purity of the isolated CD4^+^ and CD8^+^ T-cell population by positive antibody selection was determined to be between 90–95% using flow cytometry. Percentages of CD4^+^ and CD8^+^ T-cells were determined within the CD3^+^ T-cell gate and were normalized to 100.

Cell Titer 96 AQueous One Solution Cell Proliferation Assays (MTS) (Promega, Madison, WI) were performed according to the manufacturer’s protocol. Briefly, cells were counted and plated at 4,000 cells/well in 96-well round-bottom plates on day 0 and monitored over a 5-day time course. CellTiter 96 AQueous One Solution reagent was added to each well, agitated slightly, and incubated at 37°C, 5% CO_2_ for 2 hours. After 2 hours, each well was transferred to a 96-well flat-bottom plate and the absorbance at 490 nm was measured on a FilterMax F5 Multi-Mode Microplate Reader (Molecular Devices, San Jose, CA). This assay measures cellular metabolic activity as an indicator of cell viability and proliferation. Metabolic activity (i.e., proliferation) of each cell line was measured in triplicate wells at each time point.

### Nuclear/Cytoplasmic fractionation

PBL cell lines and ATL-ED cells (ED-40515) [25] were collected for nuclear/cytoplasmic fractionation using a protocol adapted from Wang et al [26]. RNA was isolated from the nuclear and cytoplasmic fractions by TRIzol extraction (Invitrogen, Carlsbad, CA). Samples were treated with DNase I recombinant, RNase-free (Roche Diagnostics GmbH, Mannheim, Germany). Nuclear or cytoplasmic RNA was used for cDNA synthesis using the SuperScript IV First-Strand Synthesis System (Invitrogen, Carlsbad, CA). 2 μL cDNA was used per qPCR reaction with iQ SYBR Green Supermix (Bio-Rad, Hercules, CA) and 300 nM of each sense and antisense primer (20 μL final volume) to detect spliced *hbz*. Primer sequences are listed in Table 2. The percentage of *hbz* localized to each fraction was determined by dividing the nuclear or cytoplasmic *hbz* copy number by the total in both fractions and normalizing to 100. Detection of *gapdh* was used to evaluate the purity of each fraction. On average, a difference of around 2 in the Cq values was observed between the nuclear and cytoplasmic fractions, where the latter exhibited a higher abundance of *gapdh*.

**Table 2:** Primer and probe sequences.

Target	Plasmid Standard	Primers & Probes	Name & Sequence
Gag/pol	ACHneo	5’ primer	[#20] 5′-^938^AGCCCCCAGTTCATGCAGACC^958^-3′
		3’ primer	[#19] 5′-^1036^GAGGGAGGAGCAAAGGTACTG^1016^-3′
		Probe	[TMP-3] 5′-/56-FAM/^990^CTGCCAAAG/ZEN/ACCTCCAAGACCTCC^1013^/3IABkFQ/-3′
Tax (transcript)	SE356	5’ primer	[X2TR1-2] 5′-^4819^ACCAACACCATGG^4831^6950^CCCA^6953^-3′
		3’ primer	[TR1-AS] 5′-^7170^GAGTCGAGGGATAAGGAAC^7152^-3′
		Probe	[TMP-2] 5’-/56-FAM/^7105^ATCACCTGGGACCCCATC^7122^/3IABkFQ/-3’
Tax (proviral load)	ACHneo	5’ outer primer	[HL43] 5’-ATGCTTATTATCAGCCCACTT-3’
		3’ outer primer	[HL44] 5’-AGGGTCTTAGAGGTTCTCTGGGT-3’
		5’ inner primer	[SK43] 5’-CGGATACCCAGTCTACGTGT-3’
		3’ inner primer	[SK44] 5’-GAGCCGATAACGCGTCCATCG-3’
		Probe	[Tax probe nested] 5’-6-FAM/ CGCCCTACTGGCCACCTGTCCAGAGCATCAGATCACCT/MGBNFQ/-3’
Hbz-365major	JA662	5’ primer	[HBZMAP1] 5′-^1905^CTTCTAAGGATAGCAAACCGTCAAG^1881^-3′
		3’ primer	[HBZMAP2] 5′-^353^ATGGCGGCCTCAG^365^1765^GGCT^1768^-3′
		Probe	[TMP-13] 5′-/56-FAM/^1782^CCTGTGCCA/ZEN/TGCCCGGAGGA^1801^/3IABkFQ/-3′
Unspliced hbz	ACHneo	cDNA synthesis	[AS2] 5′-TCTTCCTCCAAGGATAATAGCCCGTCCA-3′
		5’ primer	[AS6] 5′-CAAGGATAATAGCCCGTCCA-3′
		3’ primer	[S1] 5′-CAGTAGGGCGTGACGATGTA-3′
hGAPDH	hGAPDH	5’ primer	[hGAPDH-S] 5′-CATCAATGACCCCTTCATTGAC-3′
		3’ primer	[hGAPDH-AS] 5′-CGCCCCACTTGATTTTGGA-3′
rGAPDH	rGAPDH	5’ primer	[rGAPDH-S] 5′-GATGCTGGTGCCGAGTACGTG-3′
		3’ primer	[rGAPDH-AS] 5′- GTGGTGCAGGATGCGTTGCTGA-3
		Probe	[BY-1Z] 5′-/56-FAM/ACCACCATG/ZEN/GAGAAGGCCGGG/3IABkFQ/-3′
M3 region	N/A	5’ outer primer	[HTLV1-3LTR-1S] 5’-TCAAACCAAGGCCTACCACC-3’
		3’ outer primer	[HTLV1-3LTR-2AS] 5’-GTAACGGCGCAGAACAGAAAA-3’
		5’ inner primer	[HBZ M3 seq F] 5’-GGAGGGGGCTCGCATCTTTC-3’
		3’ inner primer	[HBZ M3 seq R] 5’-TCAGGCAAAGCGTGGAGAGC-3’
ΔHbz region	N/A	5’ outer primer	[HBZ SA seq F] 5’-CCTTTAACTCTTCCTCCAAAG-3’
		3’ outer primer	[HBZ SA seq R] 5’-GGTGGCCAGTAGGGCGTGAC-3’
		5’ inner primer	[SA nested F] 5’-GCCCGTCCACCAATTCCTCC-3’
		3’ inner primer	[SA nested R] 5’-ACCCTGGGAAGTGGGCTGAT-3’
SAm region	N/A	5’ outer primer	[HBZ SA seq F] 5’-CCTTTAACTCTTCCTCCAAAG-3’
		3’ outer primer	[HBZ SA seq R] 5’-GGTGGCCAGTAGGGCGTGAC-3’
		5’ inner primer	[SA nested F] 5’-GCCCGTCCACCAATTCCTCC-3’
		3’ inner primer	[SA nested R] 5’-ACCCTGGGAAGTGGGCTGAT-3’

### Rabbit model

Fourteen-week-old, male, specific pathogen-free New Zealand White (052 CR; 571 OAKWOOD) rabbits were obtained from Charles River Laboratories (Wilmington, MA). 10^7^ lethally irradiated (100 Gy) 729 HTLV-1 producer cells were inoculated into the lateral ear vein. Blood was drawn via the central auricular artery at Weeks 0 (pre-inoculation), 2, 4, 8, and 12 (study endpoint). Plasma was collected and rabbit PBMCs (rPBMCs) were isolated using Ficoll-Paque PREMIUM (Cytiva, Marlborough, MA). rPBMCs or plasma were assessed for proviral load, HTLV-1 gene expression, and HTLV-1-specific antibody response. Sanger sequencing was performed at Week 12 to monitor for viral reversions. The HTLV-specific antibody response was measured using the Avioq HTLV-I/II Microelisa System (Avioq, Inc., Research Triangle Park, NC). The manufacturer’s protocol was modified by substituting the provided HRP-conjugated goat anti-human IgG with an HRP-conjugated goat anti-rabbit IgG (ab6721; Abcam, Cambridge, United Kingdom).

### Humanized immune system mice

Breeding pairs of NOD.Cg-*Prkd*c^*scid*^ *Il2rg*^*tm1Wjl*^/SzJ mice (NSG strain, specific pathogen free) were purchased from The Jackson Laboratory (Bar Harbor, ME). Shortly after birth (24 to 72 h), pups were treated with whole-body irradiation at 1 Gy (RS 2000, Rad Source, Suwanee, GA). Each mouse was intrahepatically injected with 3 × 10^4^ to 1 × 10^5^ CD34^+^ human umbilical cord stem cells (HUSC) (Lonza, Allendale, NJ) in 50 μL PBS (pH 7.4). After recovery, pups were allowed to mature normally, weaned at 21 d, and then housed in groups (maximum of 5 mice per cage). At 10 wk after HUSC engraftment, mice were tested for the presence of human peripheral blood cells by flow cytometry. Mice with at least 15% human CD45-positive lymphocytes in the blood were infected by intraperitoneal inoculation of 10^6^ lethally irradiated (100 Gy) 729 HTLV-1 producer cells. At various time points after infection, aliquots of blood were mixed with commercially available fluorophore-labeled monoclonal antibodies specific for leukocyte classes: human CD4 and CD45 (BD Biosciences, Franklin Lakes, NJ). Cells and antibodies were incubated for 30 min at room temperature, and RBC were lysed using Pharm Lyse (BD Biosciences). Samples were analyzed by flow cytometry (FACSCalibur, BD Biosciences). Animals were euthanized when they lost more than 20% of their body weight within 48 h.

### Quantitative PCR

Genomic DNA and RNA were isolated from cell lines using the AllPrep DNA/RNA Mini Kit (QIAGEN, Hilden, Germany) according to manufacturer’s instructions. RNA samples were subjected to on-column DNase digestion using an RNase-Free DNase (QIAGEN, Hilden, Germany). RNA concentrations were measured using the ND-1000 Nanodrop spectrophotometer (Thermo Fisher Scientific, Waltham, MA), and 250 ng was used for cDNA synthesis using the SuperScript IV First-Strand Synthesis System (Invitrogen, Carlsbad, CA). 2 μL cDNA was used per qPCR reaction with iQ SYBR Green Supermix (Bio-Rad, Hercules, CA) and 300 nM of each sense and antisense primer (20 μL final volume). Reactions were carried out in 96-well plates on the CFX96 Touch Real-Time PCR Detection System (Bio-Rad, Hercules, CA). The reaction conditions were 50°C for 2 min, 95°C for 10 min, followed by 40 cycles of 15 sec at 95°C and 1 min at 60°C. Primer and probe sequences are listed in Table 2.

Genomic DNA and RNA were isolated from rPBMCs and HIS mouse spleens using the AllPrep DNA/RNA Mini Kit (QIAGEN, Hilden, Germany) according to manufacturer’s instructions. RNA samples were subjected to on-column DNase digestion, and 250 ng (rabbits) or 500 ng (mice) of RNA was used for cDNA synthesis, as described above. For rabbit samples only, 10 μL cDNA was pre-amplified using the SsoAdvanced PreAmp Supermix (Bio-Rad, Hercules, CA). The pre-amplification assay pool included primers for *hbz*, *gag/pol*, *tax*, and rabbit *gapdh* (*rgapdh*). The final reaction volume was 50 μL with 50 nM of each primer. The cycling protocol was as follows: 95°C for 3 min followed by 12 cycles of 95°C for 15 s and 58°C for 4 min. Products were diluted 1:5 in TE buffer (pH 8.0, RNase-free; Thermo Fisher Scientific Baltics UAB, Vilnius, Lithuania) for detection of viral genes and 1:50 for detection of high abundance targets (i.e., *rgapdh*). 8.6 μL diluted, preamplified products were used per qPCR reaction with iQ Supermix (Bio-Rad, Hercules, CA), 300 nM of each sense and antisense primer, and 100 nM probe (20 μL final reaction volume). The reaction conditions were 95°C for 3 min followed by 45 cycles of 95°C for 15 s and 57.5°C for 30 s. Total copy numbers of each gene target in cell lines, rPBMCs, and HIS mice were determined by duplicate log_10_ dilutions of plasmid DNA to generate a standard curve. Copy numbers were normalized appropriately to 1 x 10^6^ rabbit or human *gapdh*. Samples and standards were run in duplicate with no-RT and no-template controls included on each plate. To determine proviral load, 250 ng genomic DNA was used for qPCR with a primer and probe set specific to HTLV-1 *gag/pol*. The 20 μL final reaction volume included iQ Supermix (Bio-Rad, Hercules, CA), 300 nM each of 5’ primer (#20) and 3’ primer (#19), and 100 nM probe (TMP-3). The reaction conditions were as follows: 94°C for 3 min followed by 45 cycles of 94°C for 15 s, 55°C for 30 s, and 72°C for 40 s. Total copy number was calculated using a standard curve generated by duplicate log^10^ dilutions of ACHneo plasmid DNA. Proviral copies per cell were determined by estimating that 1 μg rPBMC DNA is equivalent to 134,600 cells.[27] Analysis of *gag/pol*, *tax*, and *hbz* copy numbers normalized to proviral load in rabbits was performed by first normalizing total copy number of each gene target to 1 x 10^6^ *rgapdh* and then normalizing to proviral copies per cell. Proviral copies per 100 hPBMCs in HIS mice was calculated based on the approximation that 6 pg human DNA is equivalent to 1 cell. Analysis of *tax* and *hbz* copy numbers normalized to proviral load in HIS mice was performed by first normalizing total copy number of each gene target to 1 x 10^6^ *gapdh* and then normalizing to proviral copies per 100 hPBMCs. Detection of unspliced *hbz* was performed using duplex reverse transcription followed by qPCR, as previously described [28].

### Nested PCR

The nested PCR protocol was adapted from Kwok et al [29]. Genomic DNA was isolated from rPBMCs using the AllPrep DNA/RNA Mini Kit (QIAGEN, Hilden, Germany). Up to 1 μg rabbit DNA was amplified by PCR using GoTaq Flexi DNA Polymerase (Promega, Madison, WI), GoTaq Colorless Buffer, and “outer” PCR primers listed in Table 2 targeting the *tax* gene (50 μL final reaction volume). The PCR conditions were as follows: 95°C for 90 s, 60°C for 1 min, 72°C for 1 min followed by 94°C for 1 min, 60°C for 1 min, and 72°C for 1 min (10 cycles), and a final extension of 72°C for 8 min. 8.6 μL of the outer primer PCR products were used per qPCR reaction with iQ™ Supermix (Bio-Rad, Hercules, CA), 300 nM of each “inner” primer, and 100 nM probe (20 μL final reaction volume). The reaction conditions were 95°C for 3 min followed by 40 cycles of 94°C for 1 min, 60°C for 1 min, and 72°C for 1 min. Cq values from rabbit samples were compared to values generated from log^10^ dilutions of ACHneo plasmid DNA to determine the limit of detection. Samples and standards were run in duplicate with no-template controls included on each plate.

## Results

### Generation and characterization of Hbz mutant proviral clones and producer cells

We generated the Hbz variant proviral clones in Table 1 using an established WT HTLV-1 molecular clone (ACH). The *hbz* mRNA mutant (M3) contains a single, silent point mutation that disrupts the stem-loop structure and has previously been shown to reduce the proliferative effect of the RNA *in vitro* [7]. The ΔHbz mutant includes a single G to A point mutation that severely truncates the protein at the 8^th^ amino acid but still produces spliced *hbz* mRNA [19]. The M3.ΔHbz mutant combines the M3 mutation with the ΔHbz mutation to produce *hbz* mRNA with altered secondary structure and truncated, non-functional Hbz protein. The splice acceptor mutant (SAm) harbors a T to G point mutation disrupting the *hbz* splice acceptor site [23]; thus, neither the spliced *hbz* transcript nor the protein are produced.

**Fig 1:**
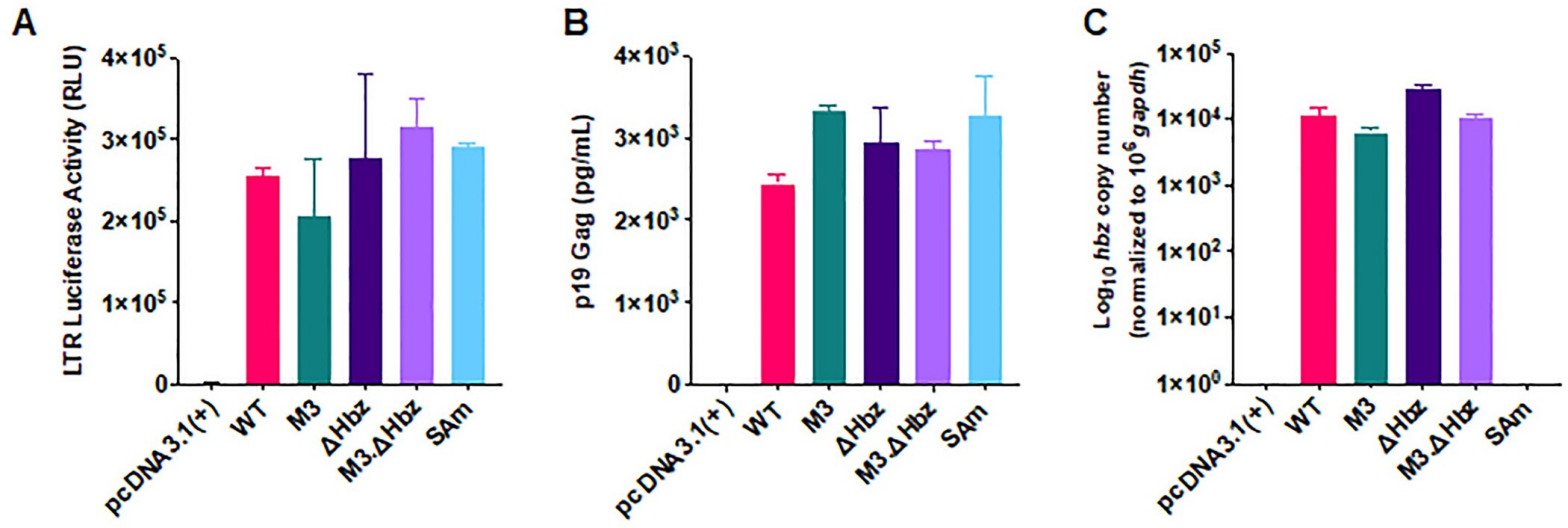
Generation and characterization of HTLV-1 *hbz* mRNA and protein mutant proviral clones in HEK293T cells. Hbz mutant constructs were generated in the context of the HTLV-1 proviral plasmid ACHneo. (A) HEK293T cells were co-transfected with pcDNA3.1(+) empty vector, WT, M3, ΔHbz, M3.ΔHbz, or SAm proviral plasmid and an HTLV-1 LTR-firefly luciferase construct. 48h post-transfection, cells and supernatant were collected for luciferase assay and ELISA to detect HTLV-1 p19 Gag (B), respectively. (C) RNA was extracted from transfected cells for cDNA synthesis and qPCR to detect *hbz* mRNA levels. *Hbz* copy number is shown normalized to 1 x 10^6^ *gapdh* copies. Graphs represent data generated from duplicate samples and error bars represent standard deviation (SD). Data are representative of at least three experimental repeats.

To determine whether the mutant proviruses were capable of Tax-mediated sense transcription, we utilized a reporter assay in HEK293T cells. There were no differences in LTR-driven luciferase activity among the mutants compared to the WT clone (Fig 1A). Further, cells transiently transfected with mutant proviral clones showed similar levels of p19 Gag in the culture supernatant to WT (Fig 1B). *Hbz* mRNA expression from each construct in transfected HEK293T cells was also quantified by RT-qPCR. The M3, ΔHbz, M3.ΔHbz mutants produced levels of *hbz* mRNA comparable to WT; however, no *hbz* spliced mRNA was detected in SAm transfected cells (Fig 1C), confirming that mutation of the *hbz* splice acceptor site successfully blocks production of the antisense viral transcript.

**Fig 2:**
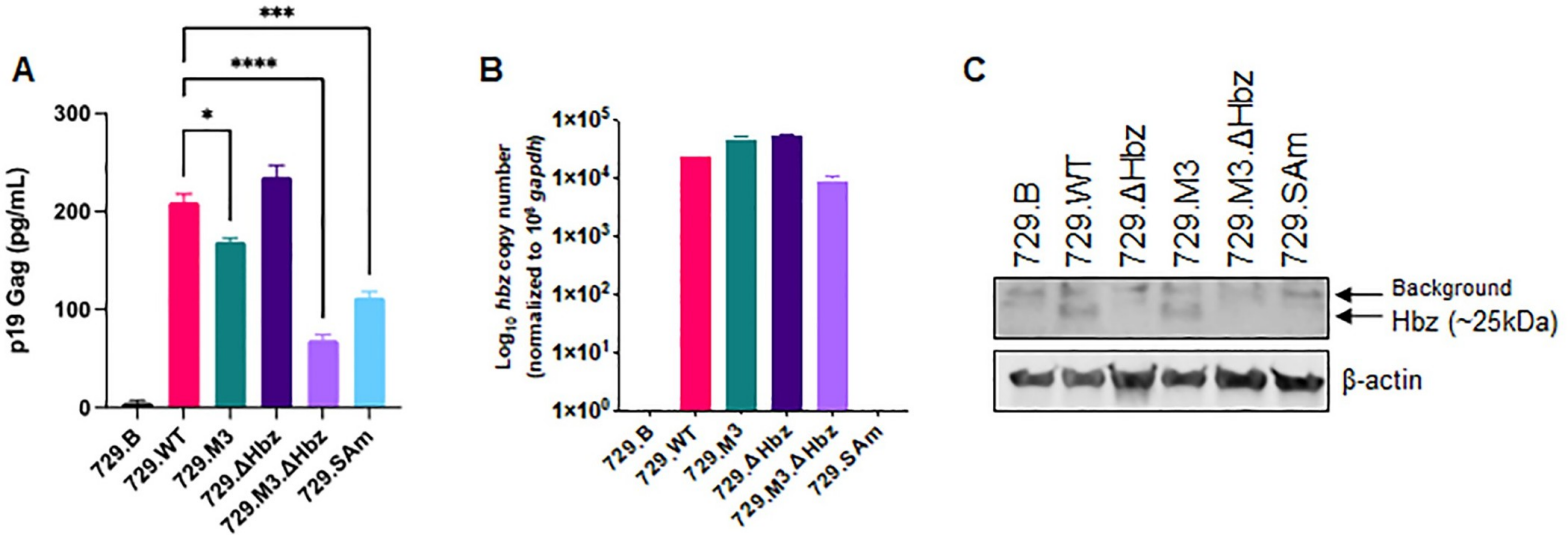
Generation and characterization of HTLV-1 *hbz* mRNA and protein mutant stable producer cell lines. WT, M3, ΔHbz, M3.ΔHbz, and SAm producer cells were generated and (A) culture supernatant was collected for p19 Gag ELISA. Statistical significance was determined by one-way ANOVA with Dunnett’s multiple comparisons test. *P ≤ 0.05, ***P ≤ 0.001, ****P ≤ 0.0001. (B) RNA was extracted from 729.B parental, WT, and mutant producer clones for cDNA synthesis and qPCR to detect *hbz* mRNA expression. *Hbz* copy number is shown normalized to 1 x 10^6^ *gapdh* copies. (C) Total protein was quantified in cell lysates from parental, WT, and mutant 729 cell lines, and equal amounts were loaded onto an SDS-PAGE gel for immunoblotting analysis. β-actin is shown as a loading control, and arrows are used to distinguish background bands from those corresponding to Hbz protein. Graphs represent data generated from duplicate samples and error bars represent SD. Data are representative of at least three experimental repeats.

Since cell-to-cell contact is required for efficient HTLV-1 transmission [30–33], we next generated stable mutant producer cell lines. After the transfection of each proviral DNA plasmid clone into 729.B parental cells, stable transfectants were isolated by antibiotic selection and limiting dilution cell cloning. The expected point mutations within each mutant were confirmed by sequencing single cell clones. Each cell line produced virus, as measured by the levels of p19 Gag in the culture supernatant (Fig 2A). While some 729.mutant cells had lower p19 Gag levels compared to 729.WT cells, this result is unsurprising due to the varying number and location of integrated viral copies between cell clones. The expression of *hbz* mRNA in each producer cell line was measured using qRT-PCR (Fig 2B). The 729.M3, 729.ΔHbz, and 729.M3.ΔHbz mutant cell lines retained the ability to synthesize *hbz* mRNA, and the transcript was undetectable in 729.SAm cells, as expected. Hbz protein expression was also evaluated and was detectable only in the 729.WT and 729.M3 conditions (Fig 2C).

### HTLV-1 *hbz* mRNA and protein mutants immortalize primary human T-cells

**Fig 3:**
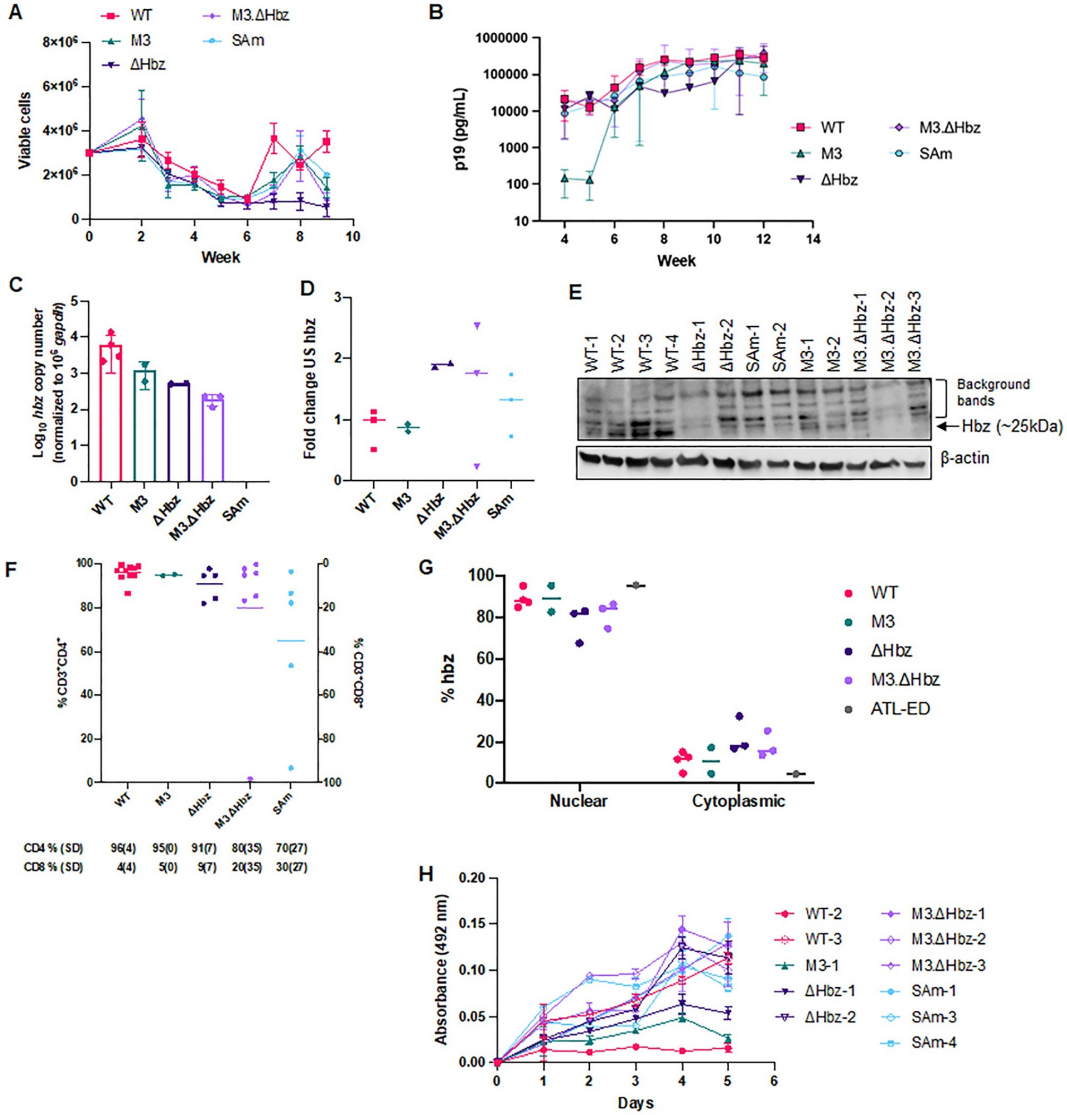
HTLV-1 *hbz* mRNA and protein mutants immortalize primary human T-cells *in vitro*. 1 x 10^6^ lethally irradiated 729.WT, 729.M3, 729.ΔHbz, 729.M3.ΔHbz, or 729.SAm producer cells were co-cultured in 24-well plates with 2 x 10^6^ freshly isolated hPBMCs from healthy donors in two independent experiments. Long-term immortalization results and characterization of newly immortalized PBL cell lines are shown from a representative experiment. (A) Cells in the co-culture were provided 10 U/mL hIL-2 once per week with fresh media. T-cell immortalization was determined with weekly viable cell counts by trypan blue exclusion. (B) Cell supernatant was collected for p19 Gag ELISA at weekly intervals, beginning at Week 4, to determine virion production. Each time point depicts data collected from three random, independent wells (technical replicates) and error bars represent SD. RNA was extracted from PBL cell lines immortalized by WT, M3, ΔHbz, M3.ΔHbz, and SAm viruses for cDNA synthesis and qPCR to detect (C) spliced *hbz* mRNA expression and (D) unspliced *hbz* RNA expression. *Hbz* copy number is shown normalized to 1 x 10^6^ *gapdh* copies. Unspliced *hbz* is shown as fold change relative to WT. Data were generated from duplicate samples and error bars represent SD. The number of newly immortalized cell lines that were analyzed for each virus are as follows: WT n = 4, M3 n = 2, ΔHbz n = 2, M3.ΔHbz n = 3, and SAm n = 3. (E) Total protein was quantified in cell lysates from WT and mutant cell lines and equal amounts were loaded onto an SDS-PAGE gel for immunoblotting analysis. β-actin is shown as a loading control, and arrows are used to distinguish background bands from those corresponding to Hbz protein. (F) Phenotyping of immortalized T-cells was performed by flow cytometry using antibodies against CD3, CD4, and CD8 surface markers. The average percentage of CD4 and CD8 T-cells in each mutant condition is depicted below. (G) Cell lines were collected for nuclear/cytoplasmic cell fractionation followed by RNA extraction, cDNA synthesis, and qPCR to detect spliced *hbz* in each fraction. Each dot represents an individual cell line, generated from duplicate samples and bars represent the mean. An ATL-derived cell line (ATL-ED) was used as a positive control. (H) Proliferation was measured by MTS assay. Error bars represent the SD of three technical replicates. Data in all panels are representative of at least three experimental repeats.

To determine the effects of *hbz* mRNA and RNA secondary structure on primary cell immortalization driven by HTLV-1, we co-cultured hPBMCs freshly isolated from healthy donors with lethally irradiated 729.WT or 729.mutant producer cells. Over time, each virus induced T-cell immortalization; despite variable rates of cell proliferation, each condition elicited immortalized, polyclonal cell populations in the late stages of the experiment (Fig 3A). As expected, PBMCs co-cultured with HTLV-1-negative 729.B parental cells did not sustain long-term proliferation. After 4 weeks, cell supernatants were collected from random wells to measure the production of p19 Gag. Although cells infected with the M3 mutant initially appeared to lag in p19 production, the levels were comparable among the WT and mutant conditions throughout the assay (Fig 3B).

Newly immortalized PBL cell lines were collected for analysis of *hbz* mRNA and protein expression. While there was some variation in *hbz* mRNA levels among the M3, ΔHbz, and M3.ΔHbz mutants compared to WT cell lines, these differences were not significant (Fig 3C). As expected, cells harboring the SAm virus lacked *hbz* mRNA expression (Fig 3C). ΔHbz, M3.ΔHbz, and SAm PBLs had no detectable Hbz protein (Fig 3E). Each of the proviral mutants theoretically should have the ability to produce unspliced *hbz* transcript, therefore newly immortalized PBL cell lines were also analyzed for *hbz* unspliced RNA transcript. Similar to *hbz* mRNA levels, there was some variation in unspliced *hbz* mRNA levels among the mutants compared to WT cell lines, however these differences were not significant and all cell lines produced unspliced *hbz* mRNA (Fig 3D). PBL cell lines immortalized by WT or Hbz mutant viruses were also phenotyped for CD3, CD4, and CD8 expression on the cell surface by flow cytometry (Fig 3F). Consistent with phenotypic data generated from previous co-culture experiments [27], our results showed that changes in *hbz* mRNA or protein production did not affect the ability of the virus to primarily immortalize CD3^+^CD4^+^ T-cells in culture.

It has recently been shown that *hbz* mRNA is inefficiently polyadenylated and retained in the nucleus of infected cells [34, 35]. Within the nucleus, *hbz* mRNA can associate with chromatin to manipulate transcription, and this function has been proposed to play a role in the persistence and pathogenesis of the virus [34]. To assess whether the Hbz mutations would affect the cellular localization of the mRNA, we conducted nuclear/cytoplasmic cell fractionation using newly immortalized HTLV-1 WT or mutant PBL cell lines. Cells immortalized by M3, ΔHbz, and M3.ΔHbz showed no significant difference in the nuclear localization of the *hbz* mRNA (Fig 3G). Finally, the proliferative capacity of WT or Hbz mutant PBLs was measured by MTS assay. Our results demonstrated that while individual cell lines had variable proliferation rates within a single condition or when compared to different groups, there were no differences that could be attributed to changes in *hbz* mRNA synthesis or structure or Hbz protein production (Fig 3H).

### Hbz protein (not mRNA) is critical for the establishment of early viral persistence *in vivo*

**Fig 4:**
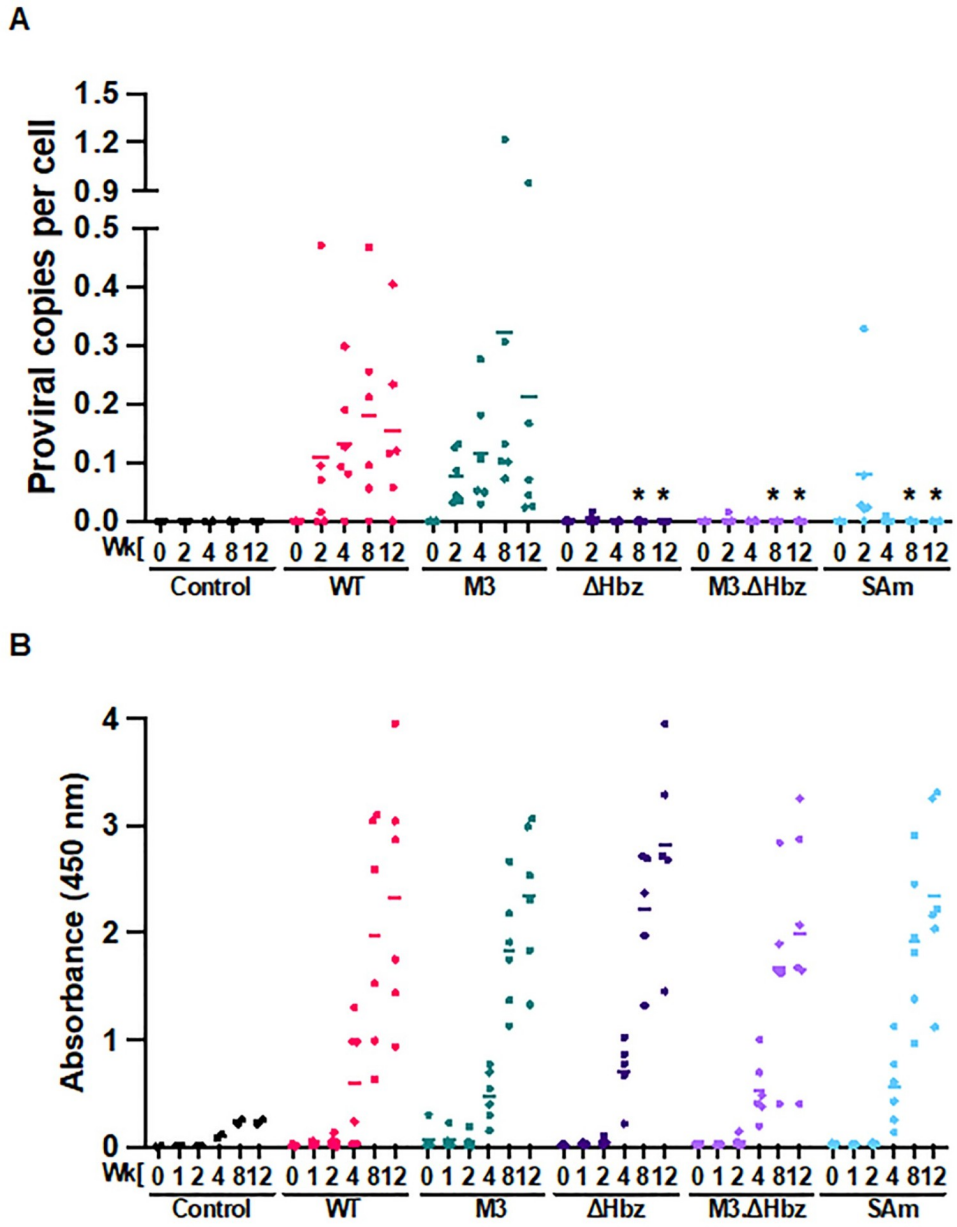
Hbz protein expression is critical for early *in vivo* viral persistence. 1 x 10^7^ lethally irradiated 729.WT, 729.M3, 729.ΔHbz, 729.M3.ΔHbz, or 729.SAm producer cells were inoculated into 14-week-old, male NZW rabbits via the lateral ear vein. Whole blood was collected at Week 0 (pre-inoculation) and Weeks 2, 4, 8, and 12 post-infection (study endpoint) for plasma and rPBMC isolation. (A) Genomic DNA was isolated from rPBMCs and proviral load was measured by qPCR using a primer and probe set specific to HTLV-1 gag/pol sequence. (B) Plasma was used to measure the HTLV-specific antibody response via the Avioq HTLV-I/II Microelisa System. Absorbances were measured at 450 nm. In each of the graphs, symbols represent the proviral load and antibody response for a single inoculated rabbit and bars represent the mean. Linear mixed-effects analyses were performed, and multiple comparisons were adjusted by Holm’s method. Asterisks represent significant differences compared to WT at the corresponding time point. *P ≤ 0.05. (Control n = 2, WT n = 6, M3 n = 6, ΔHbz n = 5, M3.ΔHbz n = 6, and SAm n = 6).

To evaluate the contributions of the *hbz* mRNA and its structure, as well as Hbz protein expression, to early viral replication events and the establishment of persistence *in vivo*, we utilized a well-characterized, immune competent NZW rabbit model of HTLV-1 infection [19, 27, 36–40]. Rabbits were inoculated with lethally irradiated 729.WT or 729.mutant producer cells, and blood was drawn at several time points over a 12-week study. In rabbits infected with WT or M3 virus, proviral copies were detectable as early as 2 weeks post-infection, and although there was variation with individual rabbits at each time point, the proviral load generally increased over the course of the study (Fig 4A). Rabbits infected with ΔHbz, M3.ΔHbz, and SAm viruses exhibited significantly decreased proviral loads at Weeks 8 and 12 compared to WT-infected rabbits. Interestingly, while low proviral copies (0.02 to 0.08 per cell) were present at Week 2 in a few rabbits in the ΔHbz, M3.ΔHbz, and SAm conditions, HTLV-1 DNA became completely undetectable by conventional qPCR in the rPBMCs from Week 4 through Week 12. To verify infection, nested PCR was performed using DNA isolated from the Week 12 time point in Table 3. Importantly, no viral reversions were detected in the mutant-infected rabbits. To assess the HTLV-1-specific immune response, rabbit plasma was analyzed by ELISA. As with previous studies, the animals had seroconverted by Week 4 post-infection, and the virus-specific response steadily increased throughout the study (Fig 4B). Surprisingly, despite the strikingly low proviral loads of ΔHbz, M3.ΔHbz, and SAm-infected rabbits, there was no significant difference in the antibody response levels among these groups compared to infected rabbits in the WT and M3 conditions (Fig 4B).

**Table 3:** Detection of HTLV-1 DNA in rPBMCs by nested PCR. Rabbits were inoculated with control (729.B), 729.WT, 729.M3, 729.ΔHbz, 729.M3.ΔHbz, or 729.SAm producer cells. Genomic DNA from rPBMCs collected and isolated 12 weeks post-infection was subjected to nested PCR with primers targeting the *tax* region.

Inoculum	Viral DNA Detected
729.B	0/2 rabbits
729.WT	6/6 rabbits
729.M3	6/6 rabbits
729.ΔHbz	5/6 rabbits
729.M3.ΔHbz	6/6 rabbits
729.SAm	6/6 rabbits

**Fig 5:**
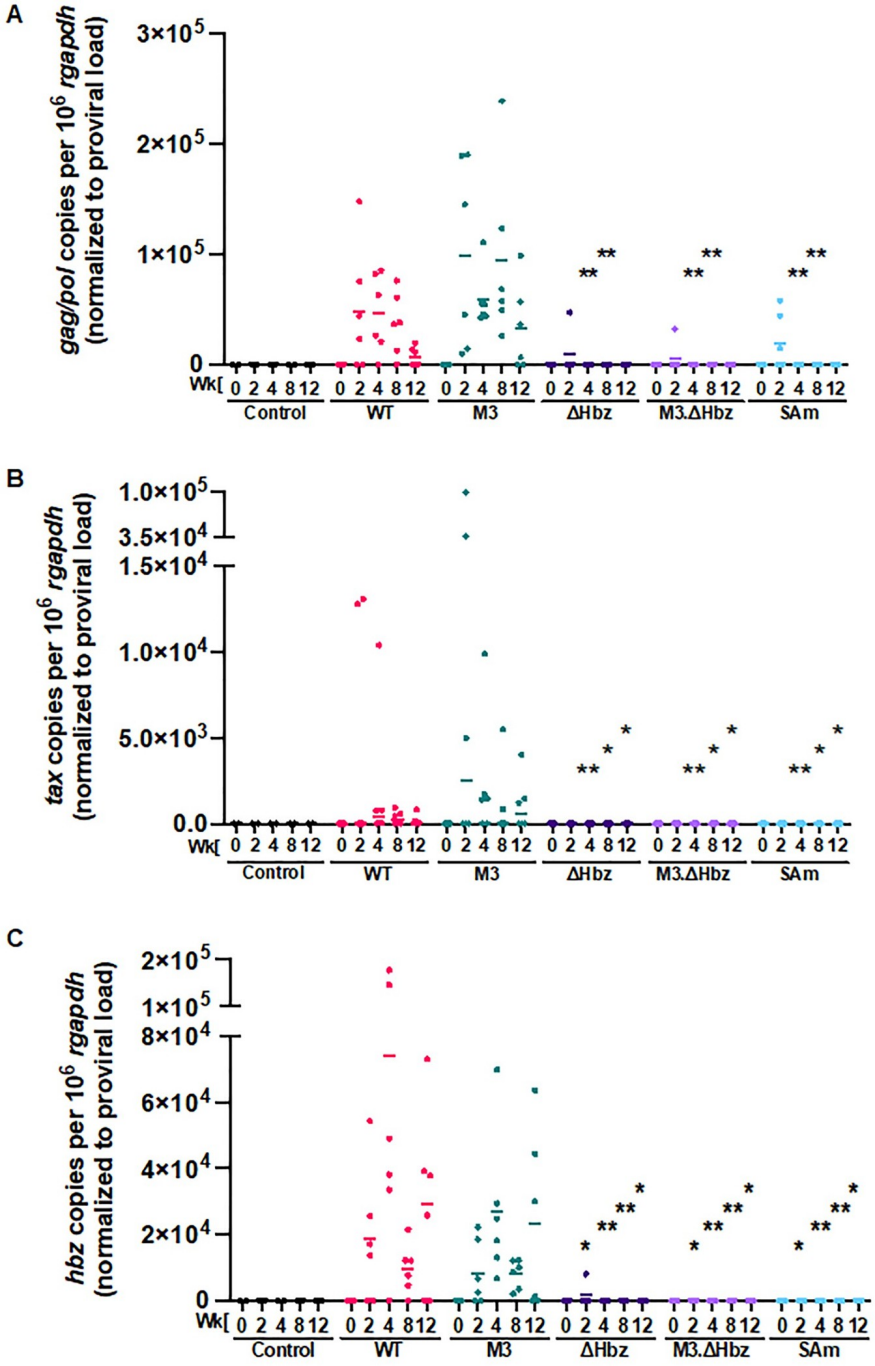
Hbz protein expression modulates levels of viral sense and antisense transcripts *in vivo*. RNA was isolated from rPBMCs for cDNA synthesis. cDNA from infected rabbits and uninfected controls was subjected to 12-cycle pre-amplification reactions using Bio-Rad SsoAdvanced™ PreAmp Supermix. Pre-amplification products were diluted according to the manufacturer’s directions for downstream qPCR to detect viral gene expression. Copy numbers of *gag/pol* (A) *tax* (B) and *hbz* (C) are shown relative to 1 x 10^6^ *rgapdh* copies. Sense and antisense viral transcripts were evaluated by normalizing copies per 10^6^ *rgapdh* to proviral load. In each of the graphs, symbols represent the gene expression for a single inoculated rabbit and bars represent the mean. Linear mixed-effects analyses were performed, and multiple comparisons were adjusted by Holm’s method. Asterisks represent significant differences compared to WT at the corresponding time point. *P ≤ 0.05, **P ≤ 0.01. (Control n = 2, WT n = 6, M3 n = 6, ΔHbz n = 5, M3.ΔHbz n = 6, and SAm n = 6).

Since Hbz can regulate sense transcription from the 5’ LTR [12], we next evaluated viral gene expression *in vivo*. Given that the levels of *tax* mRNA peak as early as 1–2 weeks post-infection [27, 38] and that this viral transcript was previously undetectable by qPCR in WT-infected rabbits [36, 41], we used *gag/pol* as a measure of sense transcription. *Gag/pol* copy number was variable at each time point in the WT and M3 conditions but plateaued at Week 8 and decreased by Week 12 (Fig 5A). Like the trends observed in proviral load (Fig 4A), *gag/pol* transcripts were detectable for the ΔHbz, M3.ΔHbz, and SAm-infected rabbits at Week 2 but decreased for the remaining weeks (Fig 5A). *Gag/pol* copies were significantly lower at Weeks 4 and 8 in the ΔHbz, M3.ΔHbz, and SAm conditions compared to WT-infected rabbits. Dynamic clonal changes 2–3 months after infection have been observed in both bovine leukemia virus (BLV) [42] and HTLV-1-infected patients [43], suggesting that *de novo* infection occurs during early stage of infection. To evaluate if loss of Hbz affects *tax* expression *in vivo*, we analyzed *tax* transcripts in the rabbits. *Tax* copy number was variable at each time point in the WT and M3 conditions but peaked quickly at Week 2 and decreased rapidly (Fig 5B). Similar to the *gag/pol* transcripts, *tax* copies were significantly lower (below the limit of detection) for the ΔHbz, M3.ΔHbz, and SAm-infected rabbits at Weeks 2, 4, 8, and 12.

*Hbz* expression was also variable among the individual WT and M3-infected rabbits at each time point, which may reflect changes in the predominance of infected cell clones over time (Fig 5C). In the ΔHbz-infected rabbits, low levels of *hbz* transcript were detected at Week 2, but not in subsequent weeks. In the M3.ΔHbz condition, *hbz* was undetectable, likely due to low proviral loads. Lastly, the SAm-infected rabbits showed no *hbz* expression, as expected (Fig 5C). Compared to WT, *hbz* copies were significantly decreased in the ΔHbz, M3.ΔHbz, and SAm conditions at Weeks 2, 4, 8, and 12. Comprehensive statistical analyses comparing each condition against the others at each time point for proviral load, antibody response, *gag/pol* expression, *tax* expression, and *hbz* expression are reported in S1–S5 Tables, respectively.

### Hbz protein expression drives leukemogenesis *in vivo*

**Fig 6:**
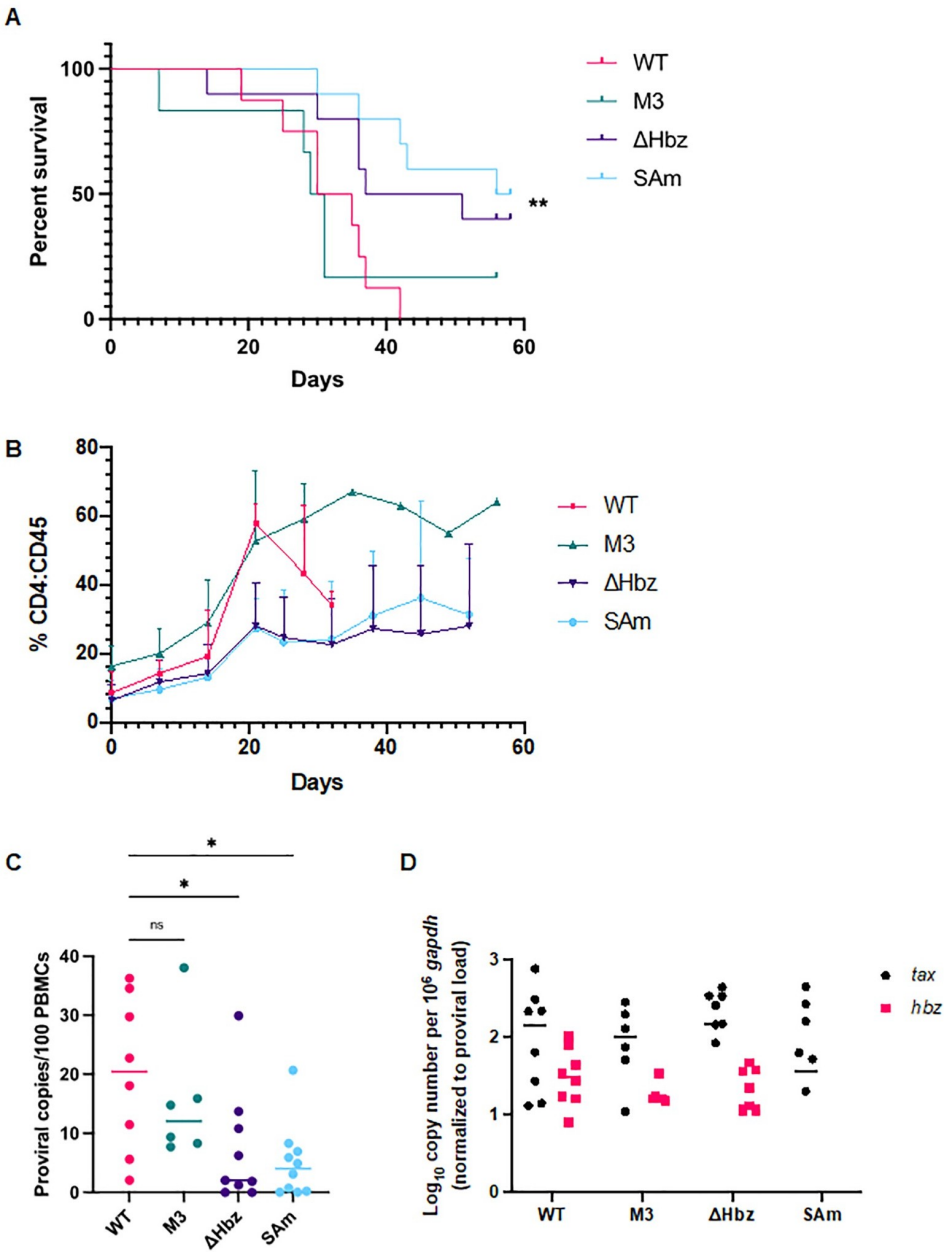
Loss of Hbz protein slows disease progression in HTLV-1-infected HIS mice. HIS mice were inoculated intraperitoneally with 1 x 10^6^ lethally irradiated 729.WT, 729.M3, 729.ΔHbz, or 729.SAm producer cells. (A) Survival rate was determined for animals inoculated with WT or mutant virus. Mice were euthanized according to early removal criteria defined in the approved animal protocol. Statistical significance was determined by Log-rank (Mantel-Cox) test. **P ≤ 0.01. (B) Development of CD4^+^ T-cells after HTLV-1 infection. The proportion of human CD4^+^ T-cells as a percentage of the total human CD45^+^ lymphocyte population is shown. (C) PBMCs were isolated from mouse spleens, and genomic DNA was extracted for qPCR to detect proviral load using primers targeting HTLV-1 *gag/pol*. Statistical significance was determined by one-way ANOVA, and multiple comparisons were adjusted by Dunnett’s method. *P ≤ 0.05. (D) RNA extracted from PBMCs was subjected to cDNA synthesis followed by qPCR to detect viral gene expression. Viral transcripts were evaluated by normalizing *tax* or *hbz* copies per 10^6^ *gapdh* to proviral load. Symbols in (B) and (C) represent proviral load and viral transcripts per proviral copy number, respectively, in a single inoculated mouse and bars represent the mean. (WT n = 9, M3 n = 6, ΔHbz n = 9, and SAm n = 10).

To determine the consequences of altered *hbz* mRNA structure or loss of mRNA or protein expression on HTLV-1-mediated disease development, we utilized humanized immune system (HIS) mice. HIS mice have previously been shown to develop lymphoproliferative disease when infected with HTLV-1 [41, 44]. These mice generate mature lymphocyte populations that are phenotypically normal but lack the capacity to mount adaptive immune responses. Throughout the study, mice were evaluated for weight loss and other clinical signs of lymphoproliferative disease induced by each virus; as the animals became moribund, they were euthanized. While there was no difference in the survival percentage between mice infected with WT or M3 virus, mice in the ΔHbz and SAm groups showed significantly increased survival times (Fig 6A). After HTLV-1 infection of HIS mice, the proportion of human CD4^+^ T-cells as a percentage of the total human CD45^+^ lymphocyte population increases very quickly [44]. Each viral mutant was capable of increasing the percentage of CD4^+^ T-cells over time (Fig 6B), an indicator of lymphoproliferative disease in the mice. The average CD4:CD45% was lower in ΔHbz and SAm groups compared to WT and M3 groups, although this difference was not statistically significant and at certain time points was based on limited numbers of mice (due to survival differences between the groups). While the mean proviral copies in M3-infected mice was lower compared to WT, the difference was not significant; however, both the ΔHbz and SAm conditions showed a significant decrease in proviral load (Fig 6C). Sequencing of genomic DNA verified the presence of the expected mutations. There were no significant differences in the level of normalized *tax* mRNA copies among the WT, M3, ΔHbz, and SAm groups (Fig 6D). In addition, normalized *hbz* mRNA copies were similar for WT, M3, and ΔHbz-infected mice.

## Discussion

The reliance of HTLV-1 on expression of antisense *hbz* gene products to support the proliferation and survival of infected cells constitutes a unique immune evasion strategy. Tax exerts important functions for the virus through its transient expression during long-term infection *in vivo* [45–47]; however, the immunogenicity of Tax and the opposing activities of Tax and Hbz promotes a dynamic interplay between the two viral oncoproteins that leads to development and progression of disease. Further, the epigenetic border separating the 5’ and 3’ regions of the integrated provirus and presence of a viral CTCF binding site suggests that active transcription from the 3’ LTR combined with remodeling of the surrounding host chromatin are major contributors to the dysregulation of an infected cell. Since the *hbz* transcript is not strongly immunogenic and its expression along with the Hbz protein is consistently detectable in ATL cells from patients, it is plausible that both mRNA and protein contribute additive effects to support the *in vivo* pathogenesis of HTLV-1. To date, the activities of the mRNA have only been studied through exogenous expression of *hbz* in HTLV-1-negative cells or methods that downregulate both mRNA and protein expression in late-stage, transformed T-cell lines [7, 20, 34]. The aims of our present study were to address the role of *hbz* mRNA and its structure during initial HTLV-1 infection of primary cells and in animal models that recapitulate viral persistence and disease progression.

In transient transfections utilizing WT and Hbz mutant proviral DNA clones, there were no differences in Tax-mediated transactivation of the 5’ LTR or virus production and release. Cells transfected with each of the mutant viruses expressed similar levels of *hbz* mRNA compared to WT (where expected). Our data from stable 729 producer cells indicated that all mutant cell lines expressed *hbz* mRNA and protein appropriately and could produce virions. In co-culture assays, each virus induced similar T-cell immortalization irrespective of the mutations that were introduced into the *hbz* gene. IL-2-dependent PBL lines newly immortalized by WT and mutant viruses exhibited similar properties in terms of cell phenotype, proliferation, and *hbz* mRNA cellular localization. Based on our results, the effects of *hbz* mRNA and protein are negligible during early stages of viral infection in our *in vitro* experimental system.

Rabbits inoculated with ΔHbz, M3.ΔHbz, and SAm viruses had significantly reduced proviral loads at various time points post-infection. In contrast, proviral copies per cell were comparable between rabbits infected with HTLV-1 WT or the M3 mutant. This suggests that the M3 point mutation disrupting the *hbz* mRNA stem-loop alone is not sufficient to reduce establishment of viral persistence. Interestingly, the results for proviral load did not coincide with a decrease in the HTLV-1-specific antibody response. This suggests that despite a pronounced reduction in viral replication in the rabbits, cells are infected and making enough viral protein to elicit an immune response. Since we only measured the antibody response through Week 12, it may not be sufficient time for antibody response to wane following initial inoculation of producer cells. In addition, we measured proviral load only in the circulating lymphocytes but do not have a clear picture of how the Hbz mutant viruses are affecting viral reservoirs. Future analysis of tissue collected from WT and Hbz mutant-infected rabbits is warranted.

HIS mice infected with ΔHbz and SAm viruses lacking the Hbz protein showed significantly increased survival times and decreases in proviral load compared to WT and M3-infected mice. Thus, the *hbz* mRNA stem-loop has no effect on HTLV-1-mediated disease development. There were no differences among the groups in normalized *tax* or *hbz* copy numbers detected in PBMCs isolated from spleens (where expected). While this provides a snapshot of viral transcript levels at the time of necropsy, it could suggest that disease progression in the infected mice is dictated by viral manipulation of host cell signaling and gene expression.

Overall, our data demonstrate that, despite the structure-dependent function that *hbz* mRNA exerts to stimulate proliferation of cell lines, alteration of a single 5’ stem-loop structure of the RNA has no effect on the ability of HTLV-1 to persist and induce leukemogenesis *in vivo*. This was the first study to investigate the contributions of the *hbz* mRNA and its 5’ stem-loop structure to viral persistence in a rabbit model and disease progression in HIS mice. However, the presence of all viral sense genes and their expression during early infection may mask the activities of the *hbz* mRNA in our experimental systems. HTLV-1 can ultimately persist for decades in an infected individual before disease symptoms appear; it is possible that *hbz* mRNA incites gradual changes that compound with the effects of Hbz and Tax proteins to promote genomic instability and the onset of malignancy. While the mechanisms remain unclear, it is likely that *hbz* mRNA alters cell signaling through interaction with cellular factors. It was reported that within the nucleus of an infected cell, the antisense transcript targets the growth-promoting *CCR4* gene, contributing to cell proliferation [34]. It has also been shown to enhance the activity of the promoter driving expression of Survivin, a member of the inhibitor of apoptosis protein family that prevents cell death [48]; thus, the *hbz* mRNA can support cell growth and survival. Further characterization of *hbz* mRNA interactions in a T-cell environment is required to elucidate its role in HTLV-1 pathogenesis.

## Supporting information

S1 Table Statistical analysis of proviral load in infected rabbits. Whole blood was collected and rPBMCs were isolated from rabbits infected with WT, M3, ΔHbz, M3.ΔHbz, or SAm viruses at Weeks 2, 4, 8, and 12 post-infection. Genomic DNA was extracted for detection of HTLV-1 proviral load by qPCR. Results of the analyses include the mean difference, standard error (SE), degrees of freedom (DF), t-value, and p-value for each comparison at each time point. The reported p-values are unadjusted and exploratory.
(DOCX)

S2 Table Statistical analysis of antibody in infected rabbits. Whole blood was collected and plasma was isolated from rabbits infected with WT, M3, ΔHbz, M3.ΔHbz, or SAm viruses at Weeks 0, 1, 2, 4, 8, and 12 post-infection. The anti-HTLV-1 antibody response was measured by ELISA. Results of the analyses include the mean difference, SE, DF, t-value, and p-value for each comparison at each time point. The reported p-values are unadjusted and exploratory.
(DOCX)

S3 Table Statistical analysis of HTLV-1 gag/pol gene expression in infected rabbits. Whole blood was collected and rPBMCs were isolated from rabbits infected with WT, M3, ΔHbz, M3.ΔHbz, or SAm viruses at Weeks 2, 4, 8, and 12 post-infection. RNA was extracted for cDNA synthesis and detection of HTLV-1 *gag/pol* gene expression by qPCR. Results of the analyses include the mean difference, SE, DF, t-value, and p-value for each comparison at each time point. The reported p-values are unadjusted and exploratory.
(DOCX)

S4 Table Statistical analysis of HTLV-1 tax gene expression in infected rabbits. Whole blood was collected and rPBMCs were isolated from rabbits infected with WT, M3, ΔHbz, M3.ΔHbz, or SAm viruses at Weeks 2, 4, 8, and 12 post-infection. RNA was extracted for cDNA synthesis and detection of HTLV-1 *tax* gene expression by qPCR. Results of the analyses include the mean difference, SE, DF, t-value, and p-value for each comparison at each time point. The reported p-values are unadjusted and exploratory.
(DOCX)

S5 Table Statistical analysis of HTLV-1 hbz gene expression in infected rabbits. Whole blood was collected and rPBMCs were isolated from rabbits infected with WT, M3, ΔHbz, M3.ΔHbz, or SAm viruses at Weeks 2, 4, 8, and 12 post-infection. RNA was extracted for cDNA synthesis and detection of HTLV-1 *hbz* gene expression by qPCR. Results of the analyses include the mean difference, SE, DF, t-value, and p-value for each comparison at each time point. The reported p-values are unadjusted and exploratory.
(DOCX)
