# Supplementary material for: HTLV-1 Hbz protein, but not hbz mRNA secondary structure, is critical for viral persistence and disease development
Source: PLoS Pathog. 2023 Jun 16;19(6):e1011459. doi: 10.1371/journal.ppat.1011459 (PMC10309998; doi:10.1371/journal.ppat.1011459)
Supplement: S1 Table — Whole blood was collected and rPBMCs were isolated from rabbits infected with WT, M3, ΔHbz, M3.ΔHbz, or SAm viruses at Weeks 2, 4, 8, and 12 post-infection. Genomic DNA was extracted for detection of HTLV-1 proviral load by qPCR. Results of the analyses include the mean difference, standard error (SE), degrees of freedom (DF), t-value, and p-value for each comparison at each time point. The reported p-values are unadjusted and exploratory. (DOCX) [file ppat.1011459.s001.docx]

**S1 Table.**

| **Condition 1** | **Week** | **Condition 2** | **Mean Difference** | **SE** | **DF** | **t-value** | **p-value** |
| --- | --- | --- | --- | --- | --- | --- | --- |
| ΔHBZ | 2 | SAm | -0.075 | 0.021 | 30.2 | -3.61 | 0.001 |
| ΔHBZ | 2 | WT | -0.104 | 0.051 | 26.4 | -2.04 | 0.051 |
| ΔHBZ | 2 | M3.ΔHBZ | 0.002 | 0.002 | 50.7 | 1.18 | 0.245 |
| ΔHBZ | 2 | M3 | -0.071 | 0.098 | 22.0 | -0.73 | 0.474 |
| WT | 2 | M3.ΔHBZ | 0.106 | 0.051 | 26.4 | 2.08 | 0.047 |
| WT | 2 | SAm | 0.029 | 0.055 | 35.0 | 0.52 | 0.603 |
| WT | 2 | M3 | 0.033 | 0.110 | 33.5 | 0.30 | 0.767 |
| M3.ΔHBZ | 2 | SAm | -0.077 | 0.021 | 30.1 | -3.71 | 0.001 |
| M3.ΔHBZ | 2 | M3 | -0.073 | 0.098 | 22.0 | -0.75 | 0.462 |
| M3 | 2 | SAm | -0.004 | 0.100 | 24.0 | -0.04 | 0.968 |
| ΔHBZ | 4 | WT | -0.132 | 0.051 | 26.4 | -2.58 | 0.016 |
| ΔHBZ | 4 | M3 | -0.116 | 0.098 | 22.0 | -1.18 | 0.249 |
| ΔHBZ | 4 | SAm | -0.002 | 0.021 | 30.2 | -0.08 | 0.937 |
| ΔHBZ | 4 | M3.ΔHBZ | 0 | 0.002 | 50.7 | 0 | 1 |
| WT | 4 | M3.ΔHBZ | 0.132 | 0.051 | 26.4 | 2.59 | 0.016 |
| WT | 4 | SAm | 0.130 | 0.055 | 35.0 | 2.36 | 0.024 |
| WT | 4 | M3 | 0.016 | 0.110 | 33.5 | 0.15 | 0.885 |
| M3.ΔHBZ | 4 | M3 | -0.116 | 0.098 | 22.0 | -1.18 | 0.249 |
| M3.ΔHBZ | 4 | SAm | -0.002 | 0.021 | 30.1 | -0.08 | 0.937 |
| M3 | 4 | SAm | 0.114 | 0.100 | 24.0 | 1.14 | 0.265 |
| ΔHBZ | 8 | WT | -0.181 | 0.051 | 26.4 | -3.55 | 0.002 |
| ΔHBZ | 8 | M3 | -0.321 | 0.098 | 22.0 | -3.29 | 0.003 |
| ΔHBZ | 8 | SAm | 0 | 0.021 | 30.2 | -0.01 | 0.994 |
| ΔHBZ | 8 | M3.ΔHBZ | 0 | 0.002 | 50.7 | 0 | 1 |
| WT | 8 | M3.ΔHBZ | 0.181 | 0.051 | 26.4 | 3.55 | 0.002 |
| WT | 8 | SAm | 0.181 | 0.055 | 35.0 | 3.29 | 0.002 |
| WT | 8 | M3 | -0.140 | 0.110 | 33.5 | -1.28 | 0.211 |
| M3.ΔHBZ | 8 | M3 | -0.321 | 0.098 | 22.0 | -3.29 | 0.003 |
| M3.ΔHBZ | 8 | SAm | 0 | 0.021 | 30.1 | -0.01 | 0.994 |
| M3 | 8 | SAm | 0.321 | 0.100 | 24.0 | 3.22 | 0.004 |
| ΔHBZ | 12 | WT | -0.155 | 0.051 | 26.4 | -3.04 | 0.005 |
| ΔHBZ | 12 | M3 | -0.213 | 0.098 | 22.0 | -2.18 | 0.040 |
| ΔHBZ | 12 | M3.ΔHBZ | 0 | 0.002 | 50.7 | 0 | 1 |
| ΔHBZ | 12 | SAm | 0 | 0.021 | 30.2 | 0 | 1 |
| WT | 12 | M3.ΔHBZ | 0.155 | 0.051 | 26.4 | 3.04 | 0.005 |
| WT | 12 | SAm | 0.155 | 0.055 | 35.0 | 2.82 | 0.008 |
| WT | 12 | M3 | -0.058 | 0.110 | 33.5 | -0.53 | 0.600 |
| M3.ΔHBZ | 12 | M3 | -0.213 | 0.098 | 22.0 | -2.18 | 0.040 |
| M3.ΔHBZ | 12 | SAm | 0 | 0.021 | 30.1 | 0 | 1 |
| M3 | 12 | SAm | 0.213 | 0.100 | 24.0 | 2.14 | 0.043 |
