# Supplementary material for: HTLV-1 Hbz protein, but not hbz mRNA secondary structure, is critical for viral persistence and disease development
Source: PLoS Pathog. 2023 Jun 16;19(6):e1011459. doi: 10.1371/journal.ppat.1011459 (PMC10309998; doi:10.1371/journal.ppat.1011459)
Supplement: S2 Table — Whole blood was collected and plasma was isolated from rabbits infected with WT, M3, ΔHbz, M3.ΔHbz, or SAm viruses at Weeks 0, 1, 2, 4, 8, and 12 post-infection. The anti-HTLV-1 antibody response was measured by ELISA. Results of the analyses include the mean difference, SE, DF, t-value, and p-value for each comparison at each time point. The reported p-values are unadjusted and exploratory. (DOCX) [file ppat.1011459.s002.docx]

**S2 Table.**

| **Condition 1** | **Week** | **Condition 2** | **Mean Difference** | **SE** | **DF** | **t-value** | **p-value** |
| --- | --- | --- | --- | --- | --- | --- | --- |
| ΔHBZ | 0 | M3 | -0.050 | 0.029 | 29 | -1.69 | 0.102 |
| ΔHBZ | 0 | M3.ΔHBZ | -0.007 | 0.029 | 29 | -0.22 | 0.825 |
| ΔHBZ | 0 | SAm | -0.006 | 0.029 | 29 | -0.20 | 0.843 |
| ΔHBZ | 0 | WT | 0.004 | 0.029 | 29 | 0.14 | 0.891 |
| WT | 0 | M3 | -0.054 | 0.028 | 29 | -1.92 | 0.065 |
| WT | 0 | M3.ΔHBZ | -0.011 | 0.028 | 29 | -0.38 | 0.707 |
| WT | 0 | SAm | -0.010 | 0.028 | 29 | -0.35 | 0.726 |
| M3.ΔHBZ | 0 | M3 | -0.043 | 0.028 | 29 | -1.54 | 0.135 |
| M3.ΔHBZ | 0 | SAm | 0.001 | 0.028 | 29 | 0.03 | 0.980 |
| M3 | 0 | SAm | 0.044 | 0.028 | 29 | 1.56 | 0.129 |
| ΔHBZ | 1 | M3 | -0.036 | 0.021 | 29 | -1.67 | 0.106 |
| ΔHBZ | 1 | SAm | 0.007 | 0.021 | 29 | 0.33 | 0.741 |
| ΔHBZ | 1 | M3.ΔHBZ | 0.004 | 0.021 | 29 | 0.18 | 0.860 |
| ΔHBZ | 1 | WT | -0.002 | 0.021 | 29 | -0.10 | 0.924 |
| WT | 1 | M3 | -0.034 | 0.020 | 29 | -1.65 | 0.110 |
| WT | 1 | SAm | 0.009 | 0.020 | 29 | 0.45 | 0.656 |
| WT | 1 | M3.ΔHBZ | 0.006 | 0.020 | 29 | 0.29 | 0.776 |
| M3.ΔHBZ | 1 | M3 | -0.040 | 0.020 | 29 | -1.94 | 0.063 |
| M3.ΔHBZ | 1 | SAm | 0.003 | 0.020 | 29 | 0.16 | 0.871 |
| M3 | 1 | SAm | 0.043 | 0.020 | 29 | 2.10 | 0.045 |
| ΔHBZ | 2 | SAm | 0.022 | 0.026 | 29 | 0.85 | 0.400 |
| ΔHBZ | 2 | M3 | -0.003 | 0.026 | 29 | -0.11 | 0.915 |
| ΔHBZ | 2 | WT | 0.002 | 0.026 | 29 | 0.09 | 0.928 |
| ΔHBZ | 2 | M3.ΔHBZ | -0.001 | 0.026 | 29 | -0.03 | 0.977 |
| WT | 2 | SAm | 0.020 | 0.025 | 29 | 0.80 | 0.431 |
| WT | 2 | M3 | -0.005 | 0.025 | 29 | -0.21 | 0.836 |
| WT | 2 | M3.ΔHBZ | -0.003 | 0.025 | 29 | -0.13 | 0.900 |
| M3.ΔHBZ | 2 | SAm | 0.023 | 0.025 | 29 | 0.93 | 0.362 |
| M3.ΔHBZ | 2 | M3 | -0.002 | 0.025 | 29 | -0.08 | 0.935 |
| M3 | 2 | SAm | 0.025 | 0.025 | 29 | 1.01 | 0.321 |
| ΔHBZ | 4 | M3 | 0.234 | 0.204 | 29 | 1.15 | 0.260 |
| ΔHBZ | 4 | M3.ΔHBZ | 0.183 | 0.204 | 29 | 0.90 | 0.376 |
| ΔHBZ | 4 | SAm | 0.157 | 0.204 | 29 | 0.77 | 0.449 |
| ΔHBZ | 4 | WT | 0.117 | 0.204 | 29 | 0.57 | 0.572 |
| WT | 4 | M3 | 0.118 | 0.195 | 29 | 0.61 | 0.549 |
| WT | 4 | M3.ΔHBZ | 0.067 | 0.195 | 29 | 0.34 | 0.734 |
| WT | 4 | SAm | 0.040 | 0.195 | 29 | 0.21 | 0.838 |
| M3.ΔHBZ | 4 | M3 | 0.051 | 0.195 | 29 | 0.26 | 0.795 |
| M3.ΔHBZ | 4 | SAm | -0.027 | 0.195 | 29 | -0.14 | 0.892 |
| M3 | 4 | SAm | -0.078 | 0.195 | 29 | -0.40 | 0.692 |
| ΔHBZ | 8 | M3.ΔHBZ | 0.543 | 0.424 | 29 | 1.28 | 0.210 |
| ΔHBZ | 8 | M3 | 0.381 | 0.424 | 29 | 0.90 | 0.376 |
| ΔHBZ | 8 | SAm | 0.300 | 0.424 | 29 | 0.71 | 0.484 |
| ΔHBZ | 8 | WT | 0.233 | 0.424 | 29 | 0.55 | 0.587 |
| WT | 8 | M3.ΔHBZ | 0.310 | 0.404 | 29 | 0.77 | 0.449 |
| WT | 8 | M3 | 0.149 | 0.404 | 29 | 0.37 | 0.716 |
| WT | 8 | SAm | 0.068 | 0.404 | 29 | 0.17 | 0.869 |
| M3.ΔHBZ | 8 | SAm | -0.243 | 0.404 | 29 | -0.60 | 0.553 |
| M3.ΔHBZ | 8 | M3 | -0.162 | 0.404 | 29 | -0.40 | 0.692 |
| M3 | 8 | SAm | -0.081 | 0.404 | 29 | -0.20 | 0.843 |
| ΔHBZ | 12 | M3.ΔHBZ | 0.832 | 0.512 | 29 | 1.62 | 0.115 |
| ΔHBZ | 12 | WT | 0.488 | 0.512 | 29 | 0.95 | 0.349 |
| ΔHBZ | 12 | M3 | 0.476 | 0.512 | 29 | 0.93 | 0.360 |
| ΔHBZ | 12 | SAm | 0.468 | 0.512 | 29 | 0.91 | 0.368 |
| WT | 12 | M3.ΔHBZ | 0.345 | 0.488 | 29 | 0.71 | 0.486 |
| WT | 12 | SAm | -0.019 | 0.488 | 29 | -0.04 | 0.969 |
| WT | 12 | M3 | -0.012 | 0.488 | 29 | -0.02 | 0.981 |
| M3.ΔHBZ | 12 | SAm | -0.364 | 0.488 | 29 | -0.75 | 0.462 |
| M3.ΔHBZ | 12 | M3 | -0.356 | 0.488 | 29 | -0.73 | 0.472 |
| M3 | 12 | SAm | -0.008 | 0.488 | 29 | -0.02 | 0.988 |
