# Supplementary material for: HTLV-1 Hbz protein, but not hbz mRNA secondary structure, is critical for viral persistence and disease development
Source: PLoS Pathog. 2023 Jun 16;19(6):e1011459. doi: 10.1371/journal.ppat.1011459 (PMC10309998; doi:10.1371/journal.ppat.1011459)
Supplement: S3 Table — Whole blood was collected and rPBMCs were isolated from rabbits infected with WT, M3, ΔHbz, M3.ΔHbz, or SAm viruses at Weeks 2, 4, 8, and 12 post-infection. RNA was extracted for cDNA synthesis and detection of HTLV-1 gag/pol gene expression by qPCR. Results of the analyses include the mean difference, SE, DF, t-value, and p-value for each comparison at each time point. The reported p-values are unadjusted and exploratory. (DOCX) [file ppat.1011459.s003.docx]

**S3 Table.**

| **Condition 1** | **Week** | **Condition 2** | **Mean Difference** | **SE** | **DF** | **t-value** | **p-value** |
| --- | --- | --- | --- | --- | --- | --- | --- |
| ΔHBZ | 2 | WT | -7.450 | 3.010 | 22.3 | -2.48 | 0.0214 |
| ΔHBZ | 2 | SAm | -4.409 | 2.073 | 44.0 | -2.13 | 0.039 |
| ΔHBZ | 2 | M3.ΔHBZ | 0.608 | 1.796 | 40.6 | 0.34 | 0.7368 |
| ΔHBZ | 2 | M3 | -12.690 | 2.065 | 42.7 | -6.15 | <.0001 |
| WT | 2 | M3.ΔHBZ | 8.058 | 2.903 | 20.0 | 2.78 | 0.0117 |
| WT | 2 | M3 | -5.240 | 3.076 | 24.0 | -1.7 | 0.1014 |
| WT | 2 | SAm | 3.041 | 3.082 | 24.2 | 0.99 | 0.3335 |
| M3.ΔHBZ | 2 | SAm | -5.017 | 1.915 | 44.2 | -2.62 | 0.012 |
| M3.ΔHBZ | 2 | M3 | -13.298 | 1.906 | 42.6 | -6.98 | <.0001 |
| M3 | 2 | SAm | 8.281 | 2.169 | 46.8 | 3.82 | 0.0004 |
| ΔHBZ | 4 | WT | -12.945 | 3.010 | 22.3 | -4.3 | 0.0003 |
| ΔHBZ | 4 | M3.ΔHBZ | 0 | 1.796 | 40.6 | 0 | 1 |
| ΔHBZ | 4 | SAm | 0 | 2.073 | 44.0 | 0 | 1 |
| ΔHBZ | 4 | M3 | -15.744 | 2.065 | 42.7 | -7.63 | <.0001 |
| WT | 4 | M3.ΔHBZ | 12.945 | 2.903 | 20.0 | 4.46 | 0.0002 |
| WT | 4 | SAm | 12.945 | 3.082 | 24.2 | 4.2 | 0.0003 |
| WT | 4 | M3 | -2.799 | 3.076 | 24.0 | -0.91 | 0.3719 |
| M3.ΔHBZ | 4 | SAm | 0 | 1.915 | 44.2 | 0 | 1 |
| M3.ΔHBZ | 4 | M3 | -15.744 | 1.906 | 42.6 | -8.26 | <.0001 |
| M3 | 4 | SAm | 15.744 | 2.169 | 46.8 | 7.26 | <.0001 |
| ΔHBZ | 8 | WT | -12.686 | 3.010 | 22.3 | -4.22 | 0.0003 |
| ΔHBZ | 8 | M3.ΔHBZ | 0 | 1.796 | 40.6 | 0 | 1 |
| ΔHBZ | 8 | SAm | 0 | 2.073 | 44.0 | 0 | 1 |
| ΔHBZ | 8 | M3 | -16.151 | 2.065 | 42.7 | -7.82 | <.0001 |
| WT | 8 | M3.ΔHBZ | 12.686 | 2.903 | 20.0 | 4.37 | 0.0003 |
| WT | 8 | SAm | 12.686 | 3.082 | 24.2 | 4.12 | 0.0004 |
| WT | 8 | M3 | -3.465 | 3.076 | 24.0 | -1.13 | 0.2712 |
| M3.ΔHBZ | 8 | SAm | 0 | 1.915 | 44.2 | 0 | 1 |
| M3.ΔHBZ | 8 | M3 | -16.151 | 1.906 | 42.6 | -8.47 | <.0001 |
| M3 | 8 | SAm | 16.151 | 2.169 | 46.8 | 7.45 | <.0001 |
| ΔHBZ | 12 | WT | -6.911 | 3.010 | 22.3 | -2.3 | 0.0314 |
| ΔHBZ | 12 | M3.ΔHBZ | 0 | 1.796 | 40.6 | 0 | 1 |
| ΔHBZ | 12 | SAm | 0 | 2.073 | 44.0 | 0 | 1 |
| ΔHBZ | 12 | M3 | -10.028 | 2.065 | 42.7 | -4.86 | <.0001 |
| WT | 12 | M3.ΔHBZ | 6.911 | 2.903 | 20.0 | 2.38 | 0.0274 |
| WT | 12 | SAm | 6.911 | 3.082 | 24.2 | 2.24 | 0.0343 |
| WT | 12 | M3 | -3.117 | 3.076 | 24.0 | -1.01 | 0.3211 |
| M3.ΔHBZ | 12 | SAm | 0 | 1.915 | 44.2 | 0 | 1 |
| M3.ΔHBZ | 12 | M3 | -10.028 | 1.906 | 42.6 | -5.26 | <.0001 |
| M3 | 12 | SAm | 10.028 | 2.169 | 46.8 | 4.62 | <.0001 |
