# Supplementary material for: HTLV-1 Hbz protein, but not hbz mRNA secondary structure, is critical for viral persistence and disease development
Source: PLoS Pathog. 2023 Jun 16;19(6):e1011459. doi: 10.1371/journal.ppat.1011459 (PMC10309998; doi:10.1371/journal.ppat.1011459)
Supplement: S4 Table — Whole blood was collected and rPBMCs were isolated from rabbits infected with WT, M3, ΔHbz, M3.ΔHbz, or SAm viruses at Weeks 2, 4, 8, and 12 post-infection. RNA was extracted for cDNA synthesis and detection of HTLV-1 tax gene expression by qPCR. Results of the analyses include the mean difference, SE, DF, t-value, and p-value for each comparison at each time point. The reported p-values are unadjusted and exploratory. (DOCX) [file ppat.1011459.s004.docx]

**S4 Table.**

| **Condition 1** | **Week** | **Condition 2** | **Mean Difference** | **SE** | **DF** | **t-value** | **p-value** |
| --- | --- | --- | --- | --- | --- | --- | --- |
| ∆HBZ | 2 | WT | -4.553 | 2.711 | 29.0 | -1.68 | 0.1038 |
| ∆HBZ | 2 | M3.∆HBZ | 0 | 2.711 | 29.0 | 0 | 1 |
| ∆HBZ | 2 | M3 | -7.335 | 2.711 | 29.0 | -2.71 | 0.0113 |
| ∆HBZ | 2 | SAm | 0 | 2.711 | 29.0 | 0 | 1 |
| WT | 2 | M3.∆HBZ | 4.553 | 2.584 | 29.0 | 1.76 | 0.0887 |
| WT | 2 | M3 | -2.783 | 2.584 | 29.0 | -1.08 | 0.2905 |
| WT | 2 | SAm | 4.553 | 2.584 | 29.0 | 1.76 | 0.0887 |
| M3.∆HBZ | 2 | M3 | -7.335 | 2.584 | 29.0 | -2.84 | 0.0082 |
| M3.∆HBZ | 2 | SAm | 0 | 2.584 | 29.0 | 0 | 1 |
| M3 | 2 | SAm | 7.335 | 2.584 | 29.0 | 2.84 | 0.0082 |
| ∆HBZ | 4 | WT | -6.392 | 2.062 | 28.0 | -3.1 | 0.0044 |
| ∆HBZ | 4 | M3.∆HBZ | 0 | 2.062 | 28.0 | 0 | 1 |
| ∆HBZ | 4 | M3 | -7.495 | 2.062 | 28.0 | -3.63 | 0.0011 |
| ∆HBZ | 4 | SAm | 0 | 2.154 | 28.0 | 0 | 1 |
| WT | 4 | M3.∆HBZ | 6.392 | 1.966 | 28.0 | 3.25 | 0.0030 |
| WT | 4 | M3 | -1.104 | 1.966 | 28.0 | -0.56 | 0.5791 |
| WT | 4 | SAm | 6.392 | 2.062 | 28.0 | 3.1 | 0.0044 |
| M3.∆HBZ | 4 | M3 | -7.495 | 1.966 | 28.0 | -3.81 | <.0001 |
| M3.∆HBZ | 4 | SAm | 0 | 2.062 | 28.0 | 0 | 1 |
| M3 | 4 | SAm | 7.495 | 2.062 | 28.0 | 3.63 | 0.0011 |
| ∆HBZ | 8 | WT | -4.631 | 1.936 | 29.0 | -2.39 | 0.0235 |
| ∆HBZ | 8 | M3.∆HBZ | 0 | 1.936 | 29.0 | 0 | 1 |
| ∆HBZ | 8 | M3 | -3.691 | 1.936 | 29.0 | -1.91 | 0.0665 |
| ∆HBZ | 8 | SAm | 0 | 1.936 | 29.0 | 0 | 1 |
| WT | 8 | M3.∆HBZ | 4.631 | 1.846 | 29.0 | 2.51 | 0.0179 |
| WT | 8 | M3 | 0.940 | 1.846 | 29.0 | 0.51 | 0.6146 |
| WT | 8 | SAm | 4.631 | 1.846 | 29.0 | 2.51 | 0.0179 |
| M3.∆HBZ | 8 | M3 | -3.691 | 1.846 | 29.0 | -2 | 0.0550 |
| M3.∆HBZ | 8 | SAm | 0 | 1.846 | 29.0 | 0 | 1 |
| M3 | 8 | SAm | 3.691 | 1.846 | 29.0 | 2 | 0.0550 |
| ∆HBZ | 12 | WT | -3.983 | 1.880 | 29.0 | -2.12 | 0.0429 |
| ∆HBZ | 12 | M3.∆HBZ | 0 | 1.880 | 29.0 | 0 | 1 |
| ∆HBZ | 12 | M3 | -5.449 | 1.880 | 29.0 | -2.9 | 0.0071 |
| ∆HBZ | 12 | SAm | 0 | 1.880 | 29.0 | 0 | 1 |
| WT | 12 | M3.∆HBZ | 3.983 | 1.793 | 29.0 | 2.22 | 0.0343 |
| WT | 12 | M3 | -1.467 | 1.793 | 29.0 | -0.82 | 0.4199 |
| WT | 12 | SAm | 3.983 | 1.793 | 29.0 | 2.22 | 0.0343 |
| M3.∆HBZ | 12 | M3 | -5.449 | 1.793 | 29.0 | -3.04 | 0.0050 |
| M3.∆HBZ | 12 | SAm | 0 | 1.793 | 29.0 | 0 | 1 |
| M3 | 12 | SAm | 5.449 | 1.793 | 29.0 | 3.04 | 0.0050 |
