# Supplementary material for: HTLV-1 Hbz protein, but not hbz mRNA secondary structure, is critical for viral persistence and disease development
Source: PLoS Pathog. 2023 Jun 16;19(6):e1011459. doi: 10.1371/journal.ppat.1011459 (PMC10309998; doi:10.1371/journal.ppat.1011459)
Supplement: S5 Table — Whole blood was collected and rPBMCs were isolated from rabbits infected with WT, M3, ΔHbz, M3.ΔHbz, or SAm viruses at Weeks 2, 4, 8, and 12 post-infection. RNA was extracted for cDNA synthesis and detection of HTLV-1 hbz gene expression by qPCR. Results of the analyses include the mean difference, SE, DF, t-value, and p-value for each comparison at each time point. The reported p-values are unadjusted and exploratory. (DOCX) [file ppat.1011459.s005.docx]

**S5 Table.**

| **Condition 1** | **Week** | **Condition 2** | **Mean Difference** | **SE** | **DF** | **t-value** | **p-value** |
| --- | --- | --- | --- | --- | --- | --- | --- |
| ΔHBZ | 2 | M3 | -6.117 | 2.241 | 37.6 | -2.73 | 0.0096 |
| ΔHBZ | 2 | WT | -7.045 | 2.828 | 17.3 | -2.49 | 0.0231 |
| ΔHBZ | 2 | M3.ΔHBZ | 2.618 | 1.152 | 20.0 | 2.27 | 0.0343 |
| ΔHBZ | 2 | SAm | 2.610 | 1.152 | 20.0 | 2.27 | 0.0348 |
| WT | 2 | M3.ΔHBZ | 9.663 | 2.583 | 12.4 | 3.74 | 0.0027 |
| WT | 2 | SAm | 9.656 | 2.583 | 12.4 | 3.74 | 0.0027 |
| WT | 2 | M3 | 0.928 | 3.219 | 25.7 | 0.29 | 0.7753 |
| M3.ΔHBZ | 2 | M3 | -8.735 | 1.922 | 23.5 | -4.55 | 0.0001 |
| M3.ΔHBZ | 2 | SAm | -0.007 | 0.022 | 46.0 | -0.34 | 0.7381 |
| M3 | 2 | SAm | 8.727 | 1.922 | 23.5 | 4.54 | 0.0001 |
| ΔHBZ | 4 | WT | -13.348 | 2.828 | 17.3 | -4.72 | 0.0002 |
| ΔHBZ | 4 | SAm | -0.025 | 1.152 | 20.0 | -0.02 | 0.9831 |
| ΔHBZ | 4 | M3.ΔHBZ | -0.012 | 1.152 | 20.0 | -0.01 | 0.9919 |
| ΔHBZ | 4 | M3 c8.10 | -14.279 | 2.241 | 37.6 | -6.37 | <.0001 |
| WT | 4 | M3.ΔHBZ | 13.336 | 2.583 | 12.4 | 5.16 | 0.0002 |
| WT | 4 | SAm | 13.323 | 2.583 | 12.4 | 5.16 | 0.0002 |
| WT | 4 | M3 | -0.931 | 3.219 | 25.7 | -0.29 | 0.7747 |
| M3.ΔHBZ | 4 | SAm | -0.013 | 0.022 | 46.0 | -0.60 | 0.5517 |
| M3.ΔHBZ | 4 | M3 | -14.267 | 1.922 | 23.5 | -7.42 | <.0001 |
| M3 | 4 | SAm | 14.254 | 1.922 | 23.5 | 7.42 | <.0001 |
| ΔHBZ | 8 | WT | -11.056 | 2.828 | 17.3 | -3.91 | 0.0011 |
| ΔHBZ | 8 | SAm | -0.048 | 1.152 | 20.0 | -0.04 | 0.9674 |
| ΔHBZ | 8 | M3.ΔHBZ | -0.030 | 1.152 | 20.0 | -0.03 | 0.9793 |
| ΔHBZ | 8 | M3 | -12.663 | 2.241 | 37.6 | -5.65 | <.0001 |
| WT | 8 | M3.ΔHBZ | 11.026 | 2.583 | 12.4 | 4.27 | 0.001 |
| WT | 8 | SAm | 11.009 | 2.583 | 12.4 | 4.26 | 0.001 |
| WT | 8 | M3 | -1.607 | 3.219 | 25.7 | -0.50 | 0.6219 |
| M3.ΔHBZ | 8 | SAm | -0.017 | 0.022 | 46.0 | -0.81 | 0.4232 |
| M3.ΔHBZ | 8 | M3 | -12.633 | 1.922 | 23.5 | -6.57 | <.0001 |
| M3 | 8 | SAm | 12.615 | 1.922 | 23.5 | 6.57 | <.0001 |
| ΔHBZ | 12 | M3 | -9.391 | 2.241 | 37.6 | -4.19 | 0.0002 |
| ΔHBZ | 12 | WT | -10.169 | 2.828 | 17.3 | -3.60 | 0.0022 |
| ΔHBZ | 12 | M3.ΔHBZ | 0.017 | 1.152 | 20.0 | 0.01 | 0.9885 |
| ΔHBZ | 12 | SAm | -0.011 | 1.152 | 20.0 | -0.01 | 0.9925 |
| WT | 12 | M3.ΔHBZ | 10.185 | 2.583 | 12.4 | 3.94 | 0.0018 |
| WT | 12 | SAm | 10.158 | 2.583 | 12.4 | 3.93 | 0.0019 |
| WT | 12 | M3 | 0.778 | 3.219 | 25.7 | 0.24 | 0.811 |
| M3.ΔHBZ | 12 | SAm | -0.028 | 0.022 | 46.0 | -1.29 | 0.2048 |
| M3.ΔHBZ | 12 | M3 | -9.408 | 1.922 | 23.5 | -4.90 | <.0001 |
| M3 | 12 | SAm | 9.380 | 1.922 | 23.5 | 4.88 | <.0001 |
